# CLIC6’s role in cancer: from broad analysis to breast cancer validation

**DOI:** 10.3389/fonc.2025.1667589

**Published:** 2025-10-09

**Authors:** Junyi Wang, Yiyang Wang, Haotian Ma, Yongxiang Li, Jiayue Hou, Jiaqi Li, Dilimulati Ismtula, Chenming Guo

**Affiliations:** ^1^ Department of Breast Surgery, Center of Digestive and Vascular, The First Affiliated Hospital of Xinjiang Medical University, Urumqi, China; ^2^ Clinical Medicine Department, Xinjiang Medical University, Urumqi, China; ^3^ The First Affiliated Hospital of Xinjiang Medical University, State Key Laboratory of Pathogenesis, Prevention, Treatment of Central Asian High Incidence Diseases in Central Asia, Urumqi, China

**Keywords:** CLIC6, pan-cancer, diagnosis, prognosis, methylation, tumor immunity

## Abstract

**Background:**

Chloride Intracellular Channel 6 (CLIC6) is a potential cancer therapy target due to its close association with tumor development. However, its diagnostic and prognostic roles, as well as its impact on immune regulation in different cancers, remain unclear.

**Methods:**

This study utilized public databases like TCGA and GEO to analyze CLIC6 expression, diagnostic value, and prognostic significance across various cancers. It examined genetic and epigenetic variations, immune correlations, and performed functional enrichment analysis to uncover CLIC6-related pathways. Western blotting confirmed CLIC6 protein levels in breast cancer samples, while CCK-8, colony formation, transwell, and scratch assays evaluated its role in cell proliferation and migration. Tissue microarray and immunohistochemistry further validated CLIC6 expression in breast cancer.

**Results:**

Research shows that CLIC6 expression is typically lower in most cancers compared to normal tissues, with distinct patterns across different stages. It serves as a useful diagnostic marker and potential prognostic factor for BRCA, LUAD, STAD, and LGG. CLIC6 mutations are common in many cancers and affect prognosis. In most tumors, CLIC6 expression correlates with m6A methylation, and its promoter is highly methylated. In BRCA, the expression of CLIC6 is related to bacterial defense, immune response, endopeptidase regulation, neuropeptide signaling, and amino acid transport. It is expressed at low levels in BRCA tissues, and we speculate that a higher CLIC6 expression may be protective.

**Conclusion:**

In conclusion, CLIC6 can serve as a key biomarker for various cancers, and its expression level is related to the tumor immune microenvironment and the outcomes in selected cancers; further validation is warranted. Our research on CLIC6 in BRCA has revealed new potential for tumor treatment strategies targeting this marker.

## 1 Introduction

Cancer is a major global health threat, with its rates of occurrence and mortality climbing each year (1). Although surgical procedures, radiotherapy, chemotherapy, and targeted therapy have significantly improved outcomes for some patients in recent years, the limitations of cancer treatment remain prominent, including drug resistance caused by tumor heterogeneity, treatment-related side effects, and poor efficacy in advanced patient (2). With the rapid development of bioinformatics and molecular biology, the discovery and application of novel biomarkers not only hold promise for optimizing existing treatment strategies but also play a crucial role in achieving early diagnosis, precise classification, treatment efficacy prediction, and prognosis assessment of cancer (3).

The chloride intracellular channel family comprises proteins that can be found in both soluble and transmembrane forms, with six homologous members. Chloride Intracellular Channel 6 (CLIC6) is the newest addition to this family, consisting of 704 amino acids in humans, and is the longest subtype identified so far (4). CLIC6 exists as a soluble protein in the cytoplasmic solute and participates in various important cellular physiological processes by regulating chloride ion transport and intracellular pH homeostasis (5). Studies have shown that CLIC6 may serve as a regulator of potential significance in cancer biology (6, 7). Additionally, CLIC6 contributes to the regulation of ion transport, signaling within cells, and the tumor microenvironment across various cellular functions (8). While earlier research has established the significance of CLIC6 in cancer, there are no comprehensive studies across all cancer types on CLIC6 at present. The study’s goal is to analyze the link between CLIC6 and the outcomes and treatment of human cancer through immunotherapy.

To illustrate the function of CLIC6 in cancer biology, this study analyzed CLIC6 using multiple databases, examined its expression levels across 33 distinct cancer types, and assessed its prognostic significance utilizing publicly available databases. Additionally, the study examined how CLIC6 is related to immune cell infiltration and conducted a functional enrichment analysis. The findings of this study contribute to understanding the role of CLIC6 in cancer and emphasize that CLIC6 influences the prognosis of cancer patients, potentially serving as a target for future treatments.

## 2 Methods

### 2.1 Analysis of CLIC6 differential expression across various cancer types

In this study, RNA sequencing data along with associated clinical information were sourced from the UCSC XENA platform (9) (https://xenabrowser.net/datapages/) for a comprehensive pan-cancer cohort comprising 15,776 samples. The dataset includes 33 different cancer sample datasets from The Cancer Genome Atlas (TCGA) and normal tissue sample datasets from the Genotype-Tissue Expression (GTEx) database (https://gtexportal.org). The RNA-seq data in the format of Transcripts Per Million (TPM) uniformly processed by the Toil process were log2 converted and used for the analysis of expression differences in subsequent studies. Data were sourced from the Gene Expression Omnibus (GEO, https://www.ncbi.nlm.nih.gov/geo/) database, including GSE7904 (platform: GPL570), GSE83889 (platform: GPL10558), GSE121248 (platform: GPL570), GSE19804 (platform: GPL570), GSE26886 (platform: GPL570), GSE54129 (platform: GPL570), GSE15471 (platform: GPL570), and GSE65144 (platform: GPL570) to validate the differential expression of CLIC6 mRNA.

To verify CLIC6 protein expression differences, initially we conducted an analysis of the protein expression and phosphorylation status of CLIC6 across various cancerous tissues and their corresponding normal tissues utilizing the “CPTAC” module available through the University of Alabama at Birmingham Cancer Data Analysis Portal (UALCAN) (10)(https://ualcan.path.uab.edu). Second, immunohistochemical images of CLIC6 protein expression in different cancers were acquired from The Human Protein Atlas (HPA) database (11)(https://www.proteinatlas.org).

### 2.2 Analysis of CLIC6’s correlation with prognosis and diagnostic value in pan-cancer

Using the median CLIC6 mRNA level, samples were split into high and low expression groups. The link between CLIC6 mRNA expression and patient outcomes for each tumor type was subsequently analyzed using Cox regression analysis. Patient prognosis information included overall survival (OS), disease-specific survival (DSS), and progression-free survival (PFS). To assess the potential value of CLIC6 in pan-cancer prognosis, we first utilized the online database Kaplan–Meier Plotter (12)(https://kmplot.com/analysis/). which is based on gene-based prognostic value meta-analyses, for analysis. Second, utilizing survival data from individual samples within the TCGA database (https://portal.gdc.cancer.gov), we analyzed the link between CLIC6 expression and prognostic indicators like OS, DSS, and PFS. in patients across various cancer types. Finally, the “ggplot2” package was used to create forest plots and Venn diagrams for visualizing results.

ROC analysis was conducted with the “pROC” package in R, and results were visualized using “ggplot2”. An Area Under the Curve (AUC) of 0.7 to 0.9 indicates that CLIC6 has moderate diagnostic potential, while an AUC of 0.9 or above signifies strong potential.

### 2.3 Creation and adjustment of prognostic nomograms

When the independent variable satisfies the proportional hazards assumption (*P*>0.05), meaning the risk associated with the independent variable does not change over time, Cox regression is used for testing. Cox proportional hazards models (both univariate and multivariate) were utilized to identify significant risk factors for patient prognosis, and factors with *P* < 0.05 were selected for inclusion in the multivariate Cox regression analysis. The cohort was split into high- and low-expression groups based on the median CLIC6 expression, and this classification was used as an independent factor. The prognostic nomogram incorporated all elements from the multivariate Cox regression analysis, and its predictive accuracy was assessed using the C-index. The analysis was conducted 1,000 times, and a calibration curve was subsequently plotted to evaluate the concordance between the predicted and actual survival outcomes. The variance inflation factor (VIF) was used to analyze whether variables in the data exhibit multicollinearity. When 0<VIF<10, no multicollinearity exists. When 10≤VIF<100, strong multicollinearity is present. When VIF≥100, multicollinearity is extremely severe.

### 2.4 Relationship between CLIC6 expression and methylation

The “TCGA” module of the UALCAN database was used to compare the CLIC6 promoter methylation levels between normal and pan-cancer samples. Additionally, the “Mutation-Methylation” module in the Cancer Gene Set Analysis Database (13, 14) (GSCA) (https://guolab.wchscu.cn/GSCA/#/) was used to explore the relationship between CLIC6 promoter methylation levels and CLIC6 expression levels, as well as the impact of CLIC6 promoter methylation levels on the prognosis (OS, PFS, DSS) of pan-cancer patients.

### 2.5 Genetic variation characteristics of CLIC6

The “Oncoprint” module of the cBioPortal database (15) (http://www.cbioportal.org/) was used to detect genetic variations in CLIC6 in the “TCGA Pan-Cancer Atlas Studies” dataset. The “Cancer Types Summary” module was used to assess the recurrence of CLIC6 gene mutations, mutation types, and copy number variations (CNVs) in each cancer type. The “Mutation” module was employed to evaluate the mutation sites of CLIC6. The percentage of CLIC6 CNVs and single nucleotide variants (SNVs) in each cancer type across all cancers was obtained from the Catalogue of Somatic Mutations in Cancer (COSMIC) database (16) (https://cancer.sanger.ac.uk/cosmic).

The association between CLIC6 CNV and CLIC6 expression levels across various cancer types was investigated using the “Mutation-CNV” module of the GSCA database, and the influence of CLIC6 CNV on the prognosis of patients with pan-cancer was evaluated.

### 2.6 Analysis of the correlation between CLIC6 expression and tumor immunity

Sangerbox3.0 (http://database.sangerbox.com/) was utilized to examine the link between CLIC6 mRNA levels and tumor mutation burden (TMB), microsatellite instability (MSI), and neoantigens (NEO) across various cancers. A radar chart illustrating these relationships was generated with the “ggplot2” package. The “estimate” package assessed the link between CLIC6 mRNA levels and tumor stromal, immune infiltration, and purity scores. To explore the link between CLIC6 expression and immune-related gene expression further, a list of immune activation and immune suppression genes was obtained from the Gene Set Enrichment Analysis (GSEA) database (17)(https://www.gsea-msigdb.org/gsea/msigdb/index.jsp), followed by Spearman correlation coefficient analysis.

Single-sample gene set enrichment analysis (ssGSEA) was performed to evaluate the association between CLIC6 mRNA levels and immune cell infiltration levels (24 cell types) across multiple cancer types. The TIMER, CIBERSORT, quanTIseq, xCell, MCP-counter, and EPIC algorithms from the “Immune” module of the Tumor Immune Estimation Resource 2.0 (TIMER 2.0) (18) (http://timer.cistrome.org/) were utilized to explore the relationship between CLIC6 mRNA expression levels and immune cell infiltration levels, including CD4+ T cells, CD8+ T cells, regulatory T cells, and B cells, in pan-cancer.

### 2.7 Functional enrichment analysis

Gene Ontology (GO)/Kyoto Encyclopedia of Genes and Genomes (KEGG) (19, 20) (https://www.kegg.jp/) enrichment analysis can predict the biological functions and related pathways involved in CLIC6. The “clusterProfiler” package and “ggplot2” package are used for functional enrichment analysis and visualization of results. The bar chart only displays the top five entries with the highest tumor-relatedness in each type.

Using the median CLIC6 mRNA expression as a cutoff, TCGA pan-cancer samples were divided into high and low expression groups. The “DESeq2” and “edgeR” packages were used to analyze differentially expressed genes, followed by GSEA analysis using the “clusterProfiler” package. The reference gene sets were obtained from the MSigDB database (21) (https://www.gsea-msigdb.org/gsea/index.jsp) under the “c2.cp.all.v2022.1.Hs.symbols.gmt” category. The analysis was performed with 5,000 iterations, and the heatmaps displayed the top 10 “Reactome” pathways for each cancer type.

### 2.8 Patient tissue sample collection

In this study, tumor and adjacent normal tissues from eight BRCA patients who underwent surgery in the Department of Breast Surgery, the First Affiliated Hospital of Xinjiang Medical University, from January 2024 to December 2024 and strictly met the inclusion and exclusion criteria (not receiving radiotherapy, chemotherapy, and hormone therapy before surgery) were collected. The Ethics Committee of the organization reviewed and approved the informed consent that patients signed prior to surgery (No. 230714-08). After surgical resection, fresh samples were quickly frozen with liquid nitrogen and stored at –80 °C before qRT-PCR and Western blotting (WB). All experimental data in this study were obtained from triplicate replicates.

### 2.9 Plasmid construction and transfection

Construction of CLIC6 knockdown vector (sh-CLIC6): Three sequences of specific small hairpin RNA (shRNA) (**Supplementary Table S1**) were selected to knock down CLIC6 expression, and the shRNA was inserted into the lentiviral vector pLKO.1-puro. Cloning was performed by restriction enzyme digestion and ligation reactions, and the constructed plasmids were verified by DNA sequencing. Construction of CLIC6 overexpression vector: Lentiviral vector pCDH was used to insert the CLIC6 gene into the appropriate position and select the appropriate promoter to drive the expression of CLIC6. The constructed vector was confirmed by DNA sequencing.

The constructed shRNA or overexpression plasmid (2 µg) was mixed with the transfection reagent Lipofectamine 3000 (Invitrogen, Shanghai, China) based on the proportion indicated by the reagent and transfected into HEK-293T cells that were grown to about 80%. The cells were cultured for 48 h to allow virus particles to be produced, thereby collecting, filtering, and concentrating the supernatant by ultracentrifugation (100,000 ×g, 4°C, 2 h). Following ultracentrifugation, the supernatant was meticulously decanted, and the resultant pellet was subsequently resuspended in a complete growth medium.

### 2.10 Lentivirus infection and grouping

MCF-7 and MDA-MB-231 breast cancer cell lines were plated, and virus infection was performed when the cells grew to about 80%. The concentrated lentivirus solution was introduced to the cells, which were replaced with fresh culture medium 24 h after infection and continued to be cultured for 48 h. According to the above methods, the cells were divided into four groups: CLIC6 knockdown empty vector group (NC-KD), CLIC6 knockdown group (KD), CLIC6 overexpression vector group (NC-OE), and CLIC6 overexpression group (OE). Gene expression was monitored using fluorescence microscopy 72 h after infection, with cells cultured under optimal conditions before being collected for further experiments.

### 2.11 RNA extraction and qRT-PCR

Following the protocols, we extracted total RNA by Trizol (Invitrogen, Shanghai, China) via the SYBR Green PCR kit (Takara, Beijing, China). Furthermore, forward and reverse primers (**Supplementary Table S1**) were used to amplify the target genes in a 20-µl final volume. The qRT-PCR reactions were conducted using the Applied Biosystems 7500 instrument (Foster City, CA, USA), and the data analysis was carried out employing the 2^–ΔΔCT^ method.

### 2.12 Protein extraction and WB

According to the protocols, protein was extracted from tissues and cells using RIPA buffer with PMSF inhibitor. The BRCA protein concentration was measured using a Beyotime assay kit from Shanghai, China. Protein samples were separated using a 10% SDS-PAGE gel and subsequently transferred onto a PVDF membrane. The membrane was then blocked and incubated with a primary antibody specific to CLIC6 (PA5-101519, Thermo Fisher, USA) at 4 °C overnight. β-Actin (Sc-69879, Santa Cruz Biotechnology, USA) served as a loading control. After a 1-h incubation at ambient temperature with secondary antibodies conjugated to horseradish peroxidase, the membrane was treated with an ECL chemiluminescent substrate (Cell Signaling Technology, Danvers, MA, USA). The resulting signals were subsequently detected using a Bio-Rad imaging system.

### 2.13 CCK-8 assay

The cell proliferation capacity was evaluated using the CCK-8 (Dojindo Molecular Technologies, Japan) by seeding cells into 96-well plates and maintaining them in culture for a duration of five days. Subsequently, the CCK-8 reagent was added to each well, followed by a 1.5-h incubation at 37 °C. The 450-nm absorbance was measured with a Tecan Infinite microplate reader (Switzerland). The measured results were plotted as a line chart to reflect the changes in cell proliferation capacity.

### 2.14 Cell clone formation assay

About 500 cells were seeded into 6-well plates and cultured in a medium with 10% fetal bovine serum (FBS) for 14 days at 37 °C with 5% CO_2_. Post-cultivation, the cells underwent fixation using 4% paraformaldehyde for 15 min, followed by staining with 0.1% crystal violet, both reagents sourced from Beyotime, Shanghai, China, for an additional 15 min. Excess dye was removed by washing with PBS, after which the colonies, defined as clusters containing more than 50 cells, were enumerated using a microscope (Leica, Germany).

### 2.15 Transwell assay

A transwell assay kit (Corning, New York, USA) was used to seed cells from the experimental and control groups into transwell chambers for invasion and migration assays, with or without Matrigel in the chambers. A 600-µl volume of 10% FBS medium was added to the lower chamber as a chemoattractant, followed by incubation for 24 h at 37 °C in 5% CO_2_. Residual cells on the upper surface of the transwell membrane were removed with a cotton swab. Cells that had traversed to the lower chamber were subsequently fixed using 4% paraformaldehyde for a duration of 30 min and stained with 0.1% crystal violet, with both reagents sourced from Beyotime, Shanghai, China. Finally, cell counts and observations were performed under an inverted microscope (Mshot, Guangzhou, China) at 200× magnification.

### 2.16 Scratch assay

To assess cell migration, scratch assays were performed. Cells were seeded in 6-well plates and grown to form a confluent monolayer. A sterile 200-µl pipette tip was used to create a uniform scratch. After detaching cells with phosphate-buffered saline (PBS), the remaining cells were cultured in a serum-free medium to reduce proliferation. Images of the scratched regions were captured at 0 and 24 h using an inverted microscope (Leica, Germany) at 100× magnification. Using ImageJ software, the scratch area was measured, and the migration rate was calculated with the following formula: (initial scratch area − final scratch area)/initial scratch area.

## 3 Statistical analysis

R software (version 4.4.2) was used to conduct statistical analyses, with data visualization facilitated by the “ggplot2” package. When the data were normally distributed with equal variances, the t-test was used. When the data were normally distributed but with unequal variances, Welch’s t-test was used. When the data were not normally distributed and required a nonparametric test, the Wilcoxon rank sum test was used. Batch correction of data was done using the Combat method. The Spearman correlation coefficient analyzed the link between CLIC6 mRNA expression and pan-cancer m6A methylation regulators, TMB, MSI, NEO, immune score, and immune-related genes. To control the false discovery rate (FDR) arising from multiple hypothesis testing across the multiple cancer types analyzed, the *P*-values were adjusted using the Benjamini–Hochberg procedure. An FDR-adjusted *P*-value of < 0.05 was considered statistically significant.

## 4 Results

### 4.1 Expression of CLIC6 in pan-cancer tissues

**Figure 1:**
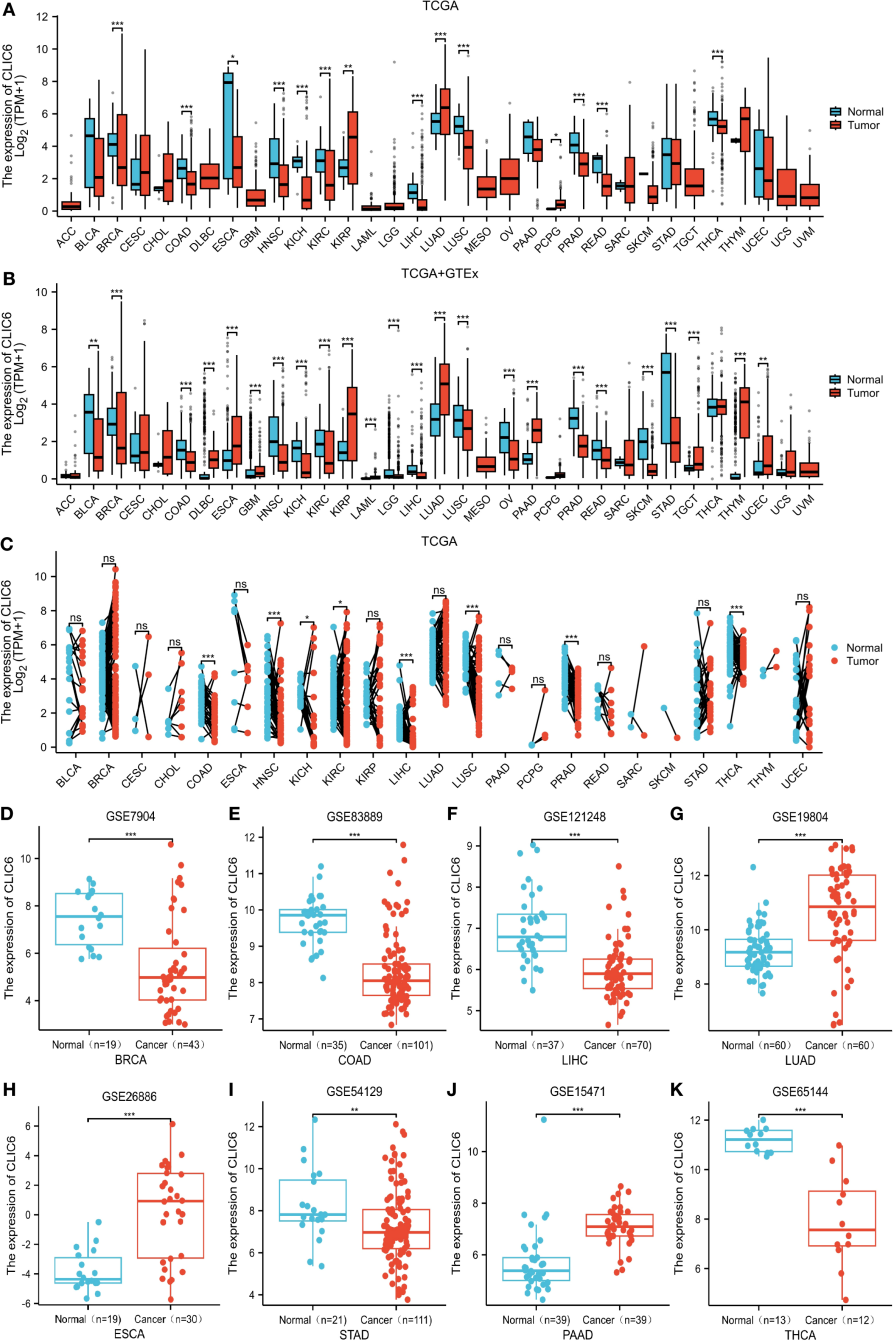
CLIC6 expression across 33 cancers. **(A)** CLIC6 mRNA levels in TCGA tumor and corresponding normal tissue. **(B)** Integration of TCGA and TCGA-GTEx for comparison of CLIC6 mRNA expression differences in normal and tumor tissues. **(C)** CLIC6 mRNA level in TCGA tumor samples vs. paired normal tissues. GEO datasets: CLIC6 expression differences for specific cancers. **(D)** BRCA (GSE7904), **(E)** COAD (GSE83889), **(F)** LIHC (GSE121248), **(G)** LUAD (GSE19804), **(H)** ESCA (GSE26886), **(I)** STAD (GSE54129), **(J)** PAAD (GSE15471), and **(K)** THCA (GSE65144) (*P<0.05, **P<0.01, ***P<0.001, NS—not significant).

First, this study assessed the expression levels of CLIC6 mRNA in various types of malignant tumors using the TCGA database. The results showed that CLIC6 mRNA expression levels were lower in 11 types of malignant tumor tissues, including BRCA, COAD, ESCA, HNSC, KICH, KIRC, LIHC, LUSC, PRAD, READ, and THCA, compared to their corresponding normal tissues (*P* < 0.05); however, CLIC6 mRNA expression levels were higher in three types of malignant tumor tissues, including KIRP, LUAD, and PCPG, in comparison to their respective healthy tissues (*P* < 0.05; **Figure 1A**).

Owing to the paucity of matched normal tissue samples for specific tumor types in TCGA, we supplemented our analysis with normal tissue data from GTEx to strengthen the robustness of our findings. The findings indicate that, compared to normal tissue, in 14 types of malignant tumor tissues, CLIC6 mRNA expression levels are notably reduced, including BLCA, BRCA, COAD, HNSC, KICH, KIRC, LGG, LIHC, LUSC, OV, PRAD, READ, SKCM, and STAD (*P* < 0.05); however, in 10 types of malignant tumor tissues, CLIC6 mRNA expression levels were notably elevated, including DLBC, ESCA, GBM, KIRP, LAML, LUAD, PAAD, TGCT, THYM, and UCEC (*P* < 0.05; **Figure 1B**).

In addition, a study of paired samples from 23 types of malignant tumors revealed that CLIC6 mRNA expression was notably reduced in eight types of malignant tumor tissues (COAD, HNSC, KICH, KIRC, LIHC, LUSC, PRAD, and THCA) compared to adjacent paracancerous tissues. However, CLIC6 mRNA expression showed no statistically significant differences in the remaining 15 paired malignant tumor-normal tissue comparisons (*P* < 0.05; **Figure 1C**).

Next, this study further validated the previous findings using datasets from the GEO database, particularly in BRCA (*P* < 0.001), COAD (*P* < 0.001), LIHC (*P* < 0.001), STAD (*P* < 0.01), and THCA (*P* < 0.001) compared to matched normal tissues; whereas CLIC6 mRNA expression levels were elevated in LUAD (*P* < 0.001), ESCA (*P* < 0.001), and PAAD (*P* < 0.001) compared to matched normal tissues (**Figures 1D-K**).

A study of the clinical and pathological features of pan-cancer in the TCGA database showed that CLIC6 expression levels were elevated in patients with early-stage BRCA, KICH, KIRP, LUAD, and THCA and gradually decreased as the tumor progressed, suggesting that CLIC6 expression may have potential value in the early diagnosis of the aforementioned cancers (**Supplementary Figures S2A-E**).

**Figure 2:**
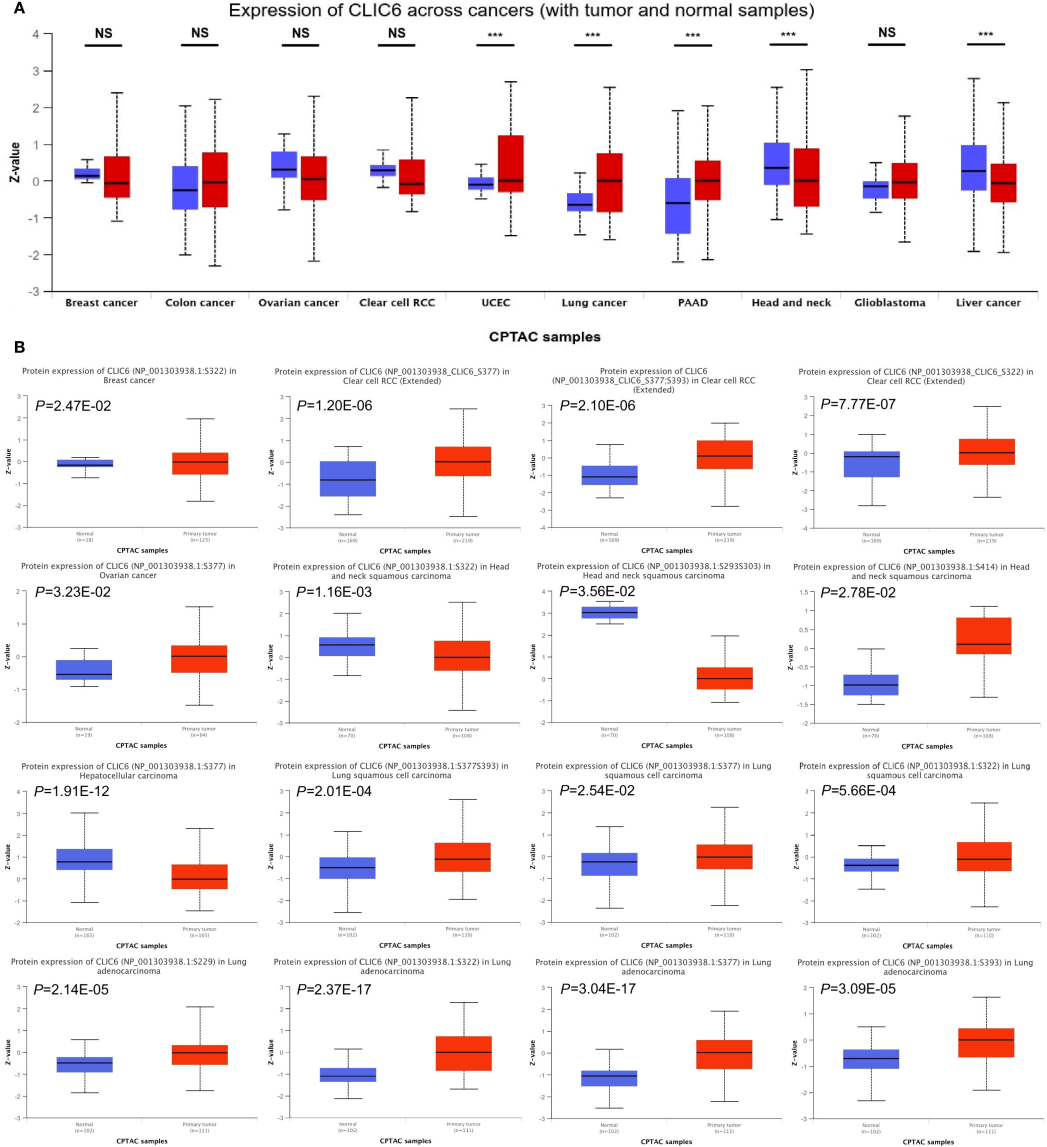
CLIC6 expression with tumor and normal samples. **(A)** CLIC6 expression across cancers (with tumor and normal samples). **(B)** CLIC6 protein level and phosphorylation sites in various cancers (***P<0.001, NS—not significant).

Next, this study used the UALCAN database to explore the expression levels of the CLIC6 protein across different cancer tissues. The results showed that CLIC6 protein levels were higher in UCEC, lung cancer, and PAAD compared to normal tissues; however, CLIC6 protein levels were lower in HNSC and liver cancer (**Figure 2A**). Additionally, changes in protein phosphorylation levels were observed in BRCA, HNSC, KIRC, LIHC, LUAD, LUSC, and OV, with the most critical phosphorylation site being NP_001303938.1:S377, followed by NP_001303938.1:S322. LUAD had the highest number of phosphorylation sites. Compared to healthy tissue, most phosphorylation sites in HNSC and LIHC exhibited reduced phosphorylation levels (**Figure 2B**).

IHC analysis from the HPA dataset showed a notable increase in CLIC6 protein expression in LUAD, GBM, PAAD, TGCT, and UCEC compared to normal tissue, aligning with previous CLIC6 mRNA findings across cancers (**Supplementary Figure S1**).

### 4.2 The prognostic and diagnostic value of CLIC6 in pan-cancer

This study analyzed the impact of CLIC6 mRNA expression levels on patient prognosis in BRCA, LUAD, COAD, STAD, and OV patients using the K-M plotter database. The findings indicated that reduced expression levels of CLIC6 mRNA were correlated with a less favorable prognosis across a majority of cancer types. This affected multiple survival metrics, such as OS, PFS, and PPS (*P* < 0.05; **Supplementary Figure S2F**).

**Figure 3:**
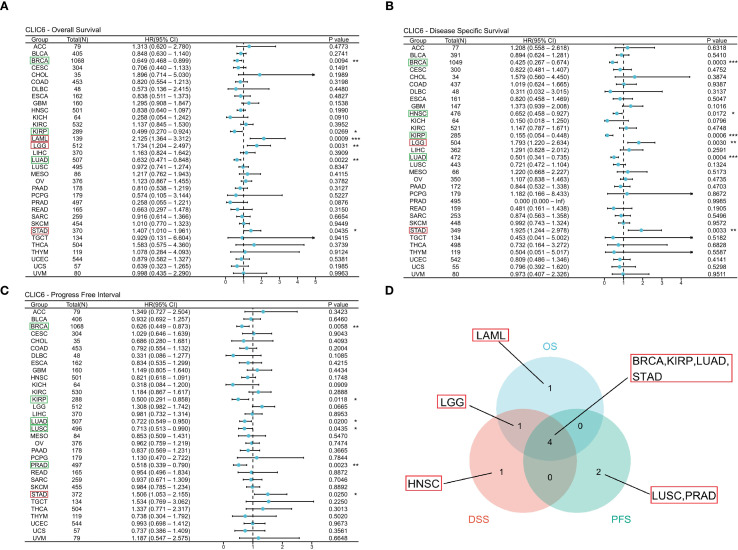
Interaction between CLIC6 expression and cancer patients’ prognosis. **(A)** Interconnection between CLIC6 expression and OS, **(B)** DSS, and **(C)** PFS. **(D)** Venn diagram: Intersection of OS, DSS, and PFS for diverse cancers (*P<0.05, **P<0.01, ***P<0.001).

This study used the TCGA database to explore the prognostic value of CLIC6 across various cancers, including three prognostic indicators (OS, DSS, and PFS). For OS, the study identified that reduced expression of CLIC6 is associated with poorer OS outcomes in patients with BRCA(*P* = 0.009, HR = 0.649), KIRP (*P* = 0.027, HR = 0.499), and LUAD (*P* = 0.002, HR = 0.632); it was an unfavorable factor for longer OS in LAML (*P* = 0.001, HR = 2.125), LGG (P = 0.003, HR = 1.734), and STAD (P = 0.043, HR = 1.407) (**Figure 3A**). For DSS, low expression of CLIC6 was an unfavorable factor for shorter DSS in patients with BRCA (P = 0.001, HR = 0.425), HNSC (P = 0.017, HR = 0.652), KIRP (P = 0.001, HR = 0.155), and LUAD (P = 0.001, HR = 0.501); it was an unfavorable factor for shorter DSS in LGG (P = 0.003, HR = 1.793) and STAD (P = 0.003, HR = 1.925) patients (**Figure 3B**). Similarly, low expression of CLIC6 is an unfavorable factor leading to shorter DSS in BRCA (P = 0.006, HR = 0.626), KIRP (P = 0.012, HR = 0.500), LUAD (P = 0.020, HR = 0.722), LUSC (P = 0.044, HR = 0.713), and PRAD (P = 0.002, HR = 0.518) patients; it is a favorable factor for longer PFS in STAD (P = 0.025, HR = 1.506) patients (**Figure 3C**). The Venn diagram results show that CLIC6 expression influences three prognostic indicators (OS, PFS, DSS) in BRCA, KIRP, LUAD, and STAD patients. This indicates that CLIC6 could be a significant determinant affecting the prognosis of these cancers (**Figure 3D**). In short, low CLIC6 expression is linked to poor outcomes in various cancers, making it a potential biomarker for predicting prognosis in pan-cancer patients.

**Figure 4:**
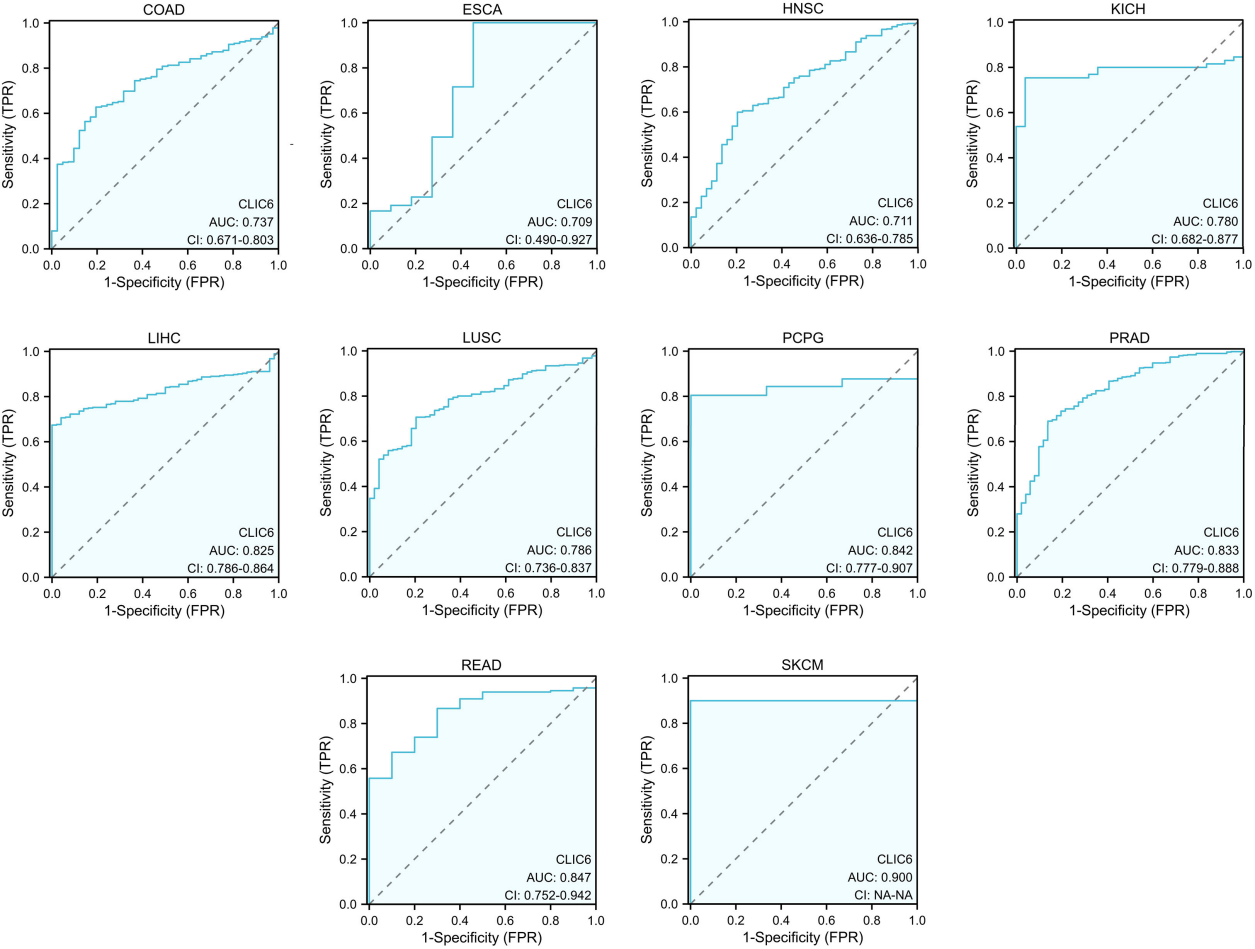
ROC curve for CLIC6 expression in pan-cancer.

**Figure 4** illustrates the additional analysis of CLIC6’s diagnostic value across various cancers in this study. The ROC curve indicates that CLIC6 is a strong diagnostic marker for SKCM (AUC≥0.9) and shows moderate diagnostic ability (AUC > 0.7) for tumors like READ, PCPG, PRAD, LIHC, LUSC, KICH, COAD, HNSC, and ESCA. Overall, CLIC6 shows moderate to high diagnostic potential in the majority of cancers.

### 4.3 CLIC6 acts as an independent factor in the prognosis of specific cancers

Prognostic factors affecting OS were systematically assessed using Cox regression modeling across four different cancer types: BRCA, LUAD, STAD, and LGG. For BRCA, the results indicated that independent predictive factors were pathological M1 stage (HR = 2.549, P = 0.005), pathological III/IV stage (HR = 2.181, P<0.001), age (>60 years, HR = 1.989, P<0.001), and CLIC6 levels (low, HR = 0.693, *P* = 0.038) (**Supplementary Table S2A**). For LUAD, pathological T3/T4 stage (HR = 1.874, *P* = 0.008), pathological N1/N2/N3 stage (HR = 2.041, P<0.001), and CLIC6 level (low, HR = 0.642, P = 0.010) represented independent predictive variables (**Supplementary Table S2B**). For STAD, primary treatment outcomes (disease progression (PD)/disease stabilization (SD), HR = 0.267, P<0.001), age (>65 years, HR = 1.754, P = 0.007), and CLIC6 levels (high, HR = 1.701, P = 0.010) represented independent predictive variables (**Supplementary Table S2C**). For LGG, WHO grade (G3, HR = 2.748, P<0.001), PD/SD (HR = 0.209, P<0.001), age (>40 years, HR = 2.881, P<0.001), and CLIC6 levels (high, HR = 1.978, P = 0.001) represented independent predictive variables (**Supplementary Table S2D**).

**Figure 5:**
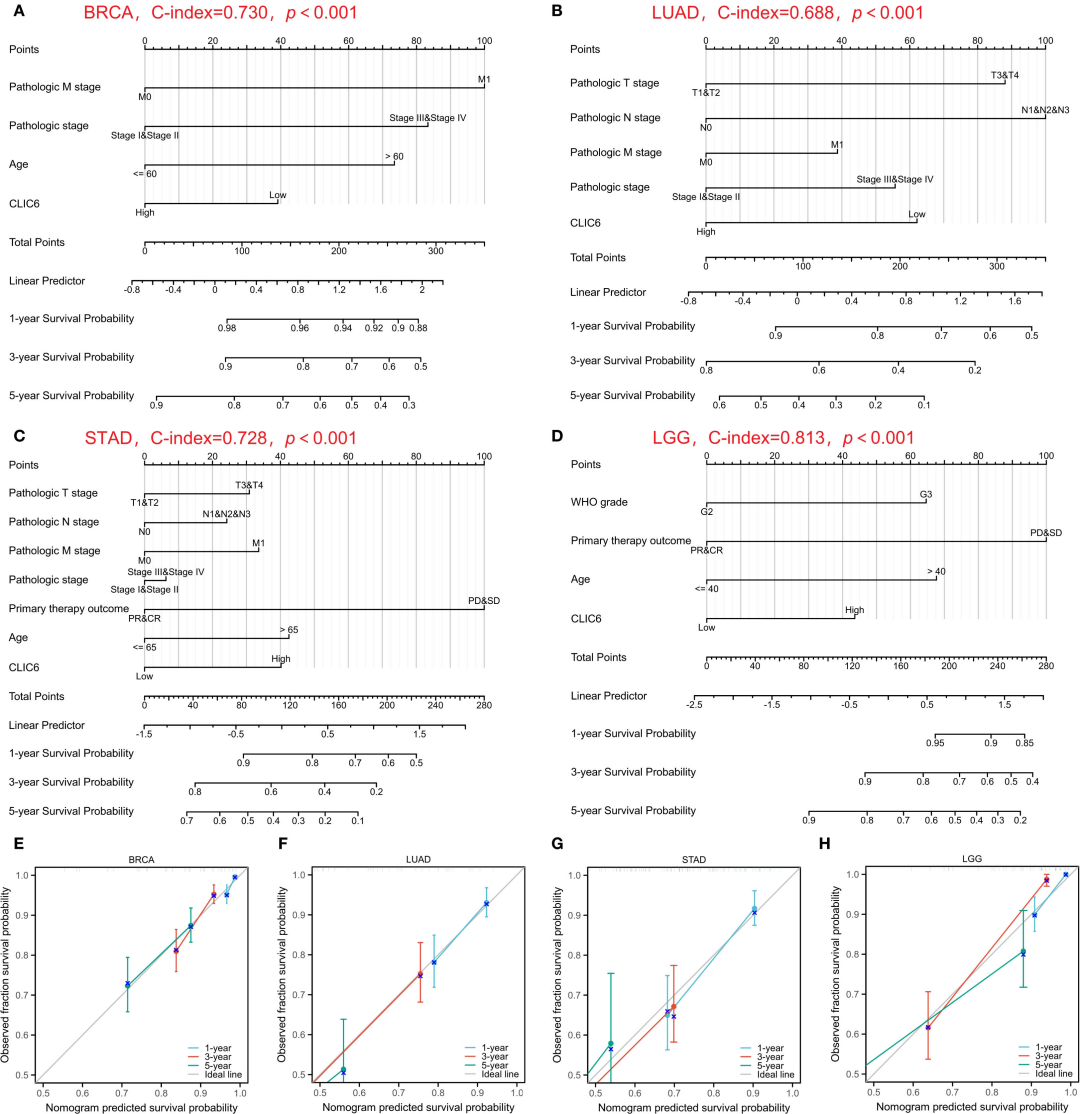
Nomograms prediction and calibration curve of patient OS in four cancers. Nomograms for BRCA **(A)**, LUAD **(B)**, STAD **(C)**, LGG **(D)**. Calibration curve for BRCA **(E)**, LUAD **(F)**, STAD **(G)**, LGG **(H)**.

Subsequently, nomogram predictors were selected based on univariate significance (*P* < 0.1), with model accuracy subsequently verified through calibration plotting. The results showed that the C-index of the prognostic nomogram for BRCA was 0.730 (0.707–0.753), and the C-index for LUAD was 0.688 (0.663–0.712). The C-index for STAD was 0.728 (0.703–0.752), and the C-index for LGG was 0.813 (0.792–0.834) (**Figures 5A-D**). Furthermore, all developed nomograms exhibited excellent calibration accuracy across the spectrum of predicted probabilities for the four cancer types (**Figures 5E-H**). Therefore, CLIC6 demonstrates independent predictive value for patient survival in these cancer types.

### 4.4 Genetic variation characteristics of CLIC6 in pan-cancer

**Figure 6:**
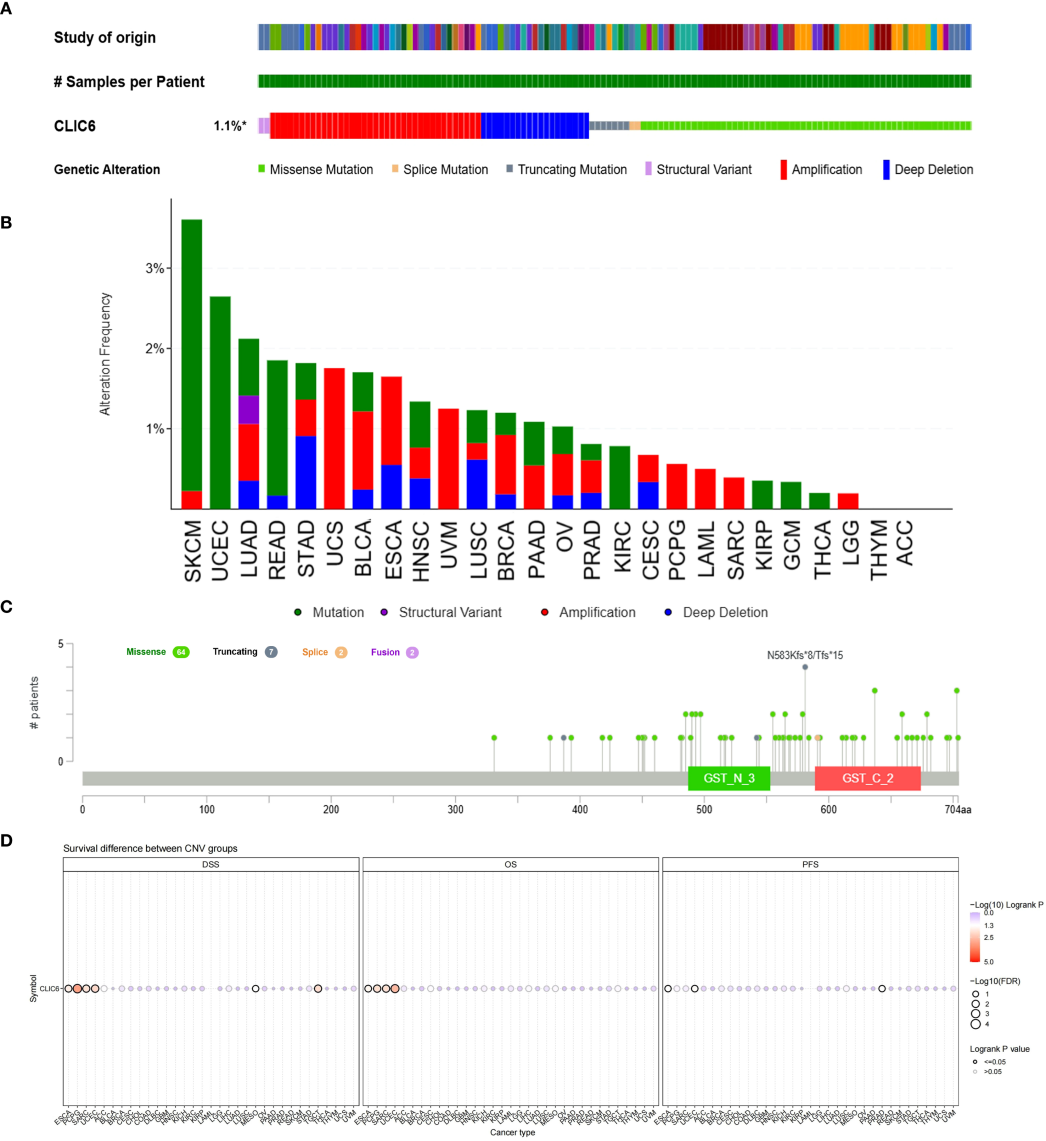
Mutational landscape of CLIC6 in cancer (n=10,967) **(A)** Expression alterations across tumors. **(B, C)** Mutation distribution and mapped sites. **(D)** Prognostic impact of CLIC6 CNVs.

The development of cancer is influenced by various genetic alterations, among which there are key factors that could serve as potential targets for molecular therapy (22). To explore whether CLIC6 could be a molecular therapy target, this study used the cBioPortal database to investigate CLIC6 genetic variation across various cancer types. Results showed that among 10,967 samples, 121 samples (1.1%) harbored CLIC6 mutations. Amplification was the most common CNV mutation, followed by missense mutations, deep deletions, truncation mutations, and splicing mutations (**Figure 6A**). Among these, missense mutations accounted for 34.46%, and synonymous substitutions accounted for 14.98% (**Supplementary Figure S4A**). Moreover, the most common SNV category was G>A (36.75%), followed by C>T (23.51%) (**Supplementary Figure S4B**). Subsequently, the mutation status of the CLIC6 gene was investigated across various cancer types. Among various cancer types, SKCM (3.62%), UCEC (2.65%), LUAD (2.12%), READ (1.85%), and STAD (1.82%) exhibited the highest mutation frequencies (**Figure 6B**). N583Kfs*8/Tfs*15 was the most frequently mutated locus within the CLIC6 domain (**Figure 6C**).

Subsequently, the relationship between CLIC6 mutations and CLIC6 mRNA expression, as well as their association with prognosis in pan-cancer patients, was investigated using the GSCA database. The CNV pie chart derived from this database showed that most cancers exhibited heterozygous amplification and deletion, and homozygous amplification, with rare homozygous deletions occasionally occurring in BRCA, PRAD, CESC, LUAD, COAD, HNSC, STAD, LUSC, and ESCA (**Supplementary Figure S4C**). Additionally, in cancers such as SKCM, LUSC, LUAD, PAAD, and HNSC, the occurrence of CNVs in CLIC6 was positively correlated with CLIC6 mRNA expression levels (*P* < 0.05; **Supplementary Figure S4D**). Furthermore, this study found that CLIC6 CNV is an important factor contributing to poor prognosis (OS, DSS) in patients with ESCA, PCPG, SARC, and UCEC (**Figure 6D**). In summary, CLIC6 genetic alterations occur in most malignant tumors and influence cancer patient prognosis. Additionally, genetic alterations in CLIC6 could represent viable targets for molecular therapeutic interventions.

### 4.5 Exploring the association between CLIC6 and methylation

**Figure 7:**
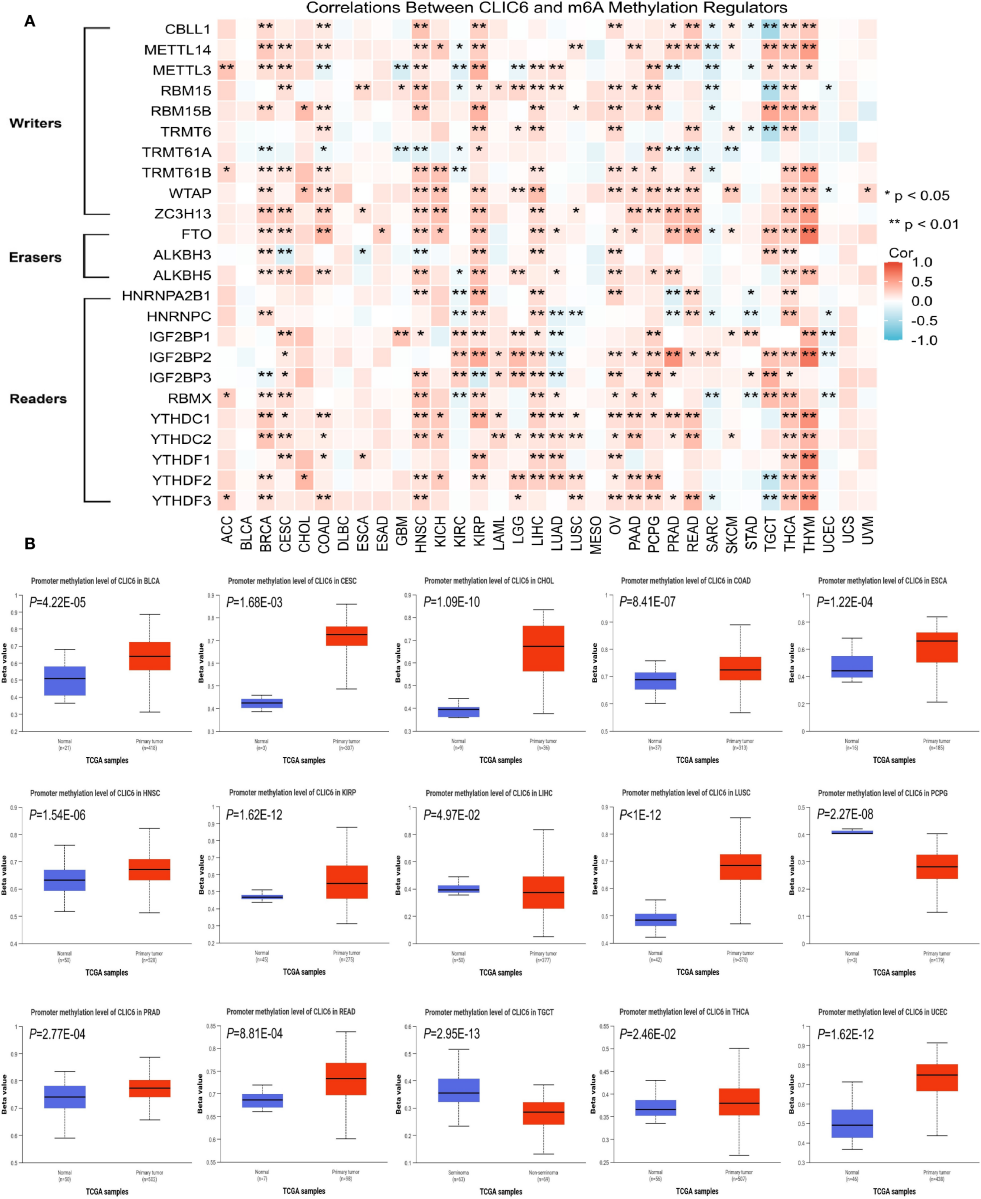
Epigenetic methylation analysis of CLIC6. **(A)** CLIC6-m6A regulator interactions across cancers. **(B)** Differential promoter methylation in tumors vs. normal tissues.

Earlier research indicates that m6A methylation plays a significant role in the metabolic reprogramming of tumor cells, influencing tumor development and progression by altering tumor metabolism (23). Therefore, this study further explored the interplay between CLIC6 mRNA expression and key m6A methylation regulators in certain cancers. A total of 24 m6A methylation regulators were selected: 10 writers, 3 erasers, and 11 readers. Most tumors show a positive correlation between CLIC6 expression and m6A methylation regulator expression in heatmaps (**Figure 7A**).

Additionally, promoter methylation profiles of CLIC6 in tumor and matched normal tissues were analyzed using the UALCAN platform. The CpG probe IDs used were cg15295166, cg11528328, cg08302532, cg19200589, cg18074297, cg20343048, cg16057826, cg16628066, and cg18371700. The data demonstrated that, compared to healthy tissues, the CLIC6 promoter exhibited significantly higher methylation levels in BLCA (*P* = 4.22E-05), CESC (*P* = 1.68E-03), CHOL (*P* = 1.09E-10), COAD (*P* = 8.41E-07), ESCA (*P* = 1.22E-04), HNSC (*P* = 1.54E-06), KIRP (*P* = 1.62E-12), LUSC (*P*<1E-12), PRAD (*P* = 2.77E-04), READ (*P* = 8.81E-04), THCA (*P* = 2.46E-02), and UCEC (*P* = 1.62E-12); in contrast, methylation levels were lower in malignant tumor tissues such as LIHC (*P* = 4.97E-02), PCPG (*P* = 2.27E-08), and TGCT (*P* = 2.95E-13) (**Figure 7B**). Subsequently, the GSCA database was used to analyze the impact of CLIC6 promoter methylation levels on cancer patient outcomes, including OS, PFS, and DSS. The results showed that high CLIC6 promoter methylation was a protective factor associated with better outcomes in LGG patients; it was an adverse factor associated with poorer OS and DSS in ACC patients (*P* < 0.05, **Supplementary Figure S4E**).

To sum up, the CLIC6 promoter is highly methylated in the majority of cancers and influences patient outcomes.

### 4.6 Analysis of the correlation between CLIC6 expression and immunity

**Figure 8:**
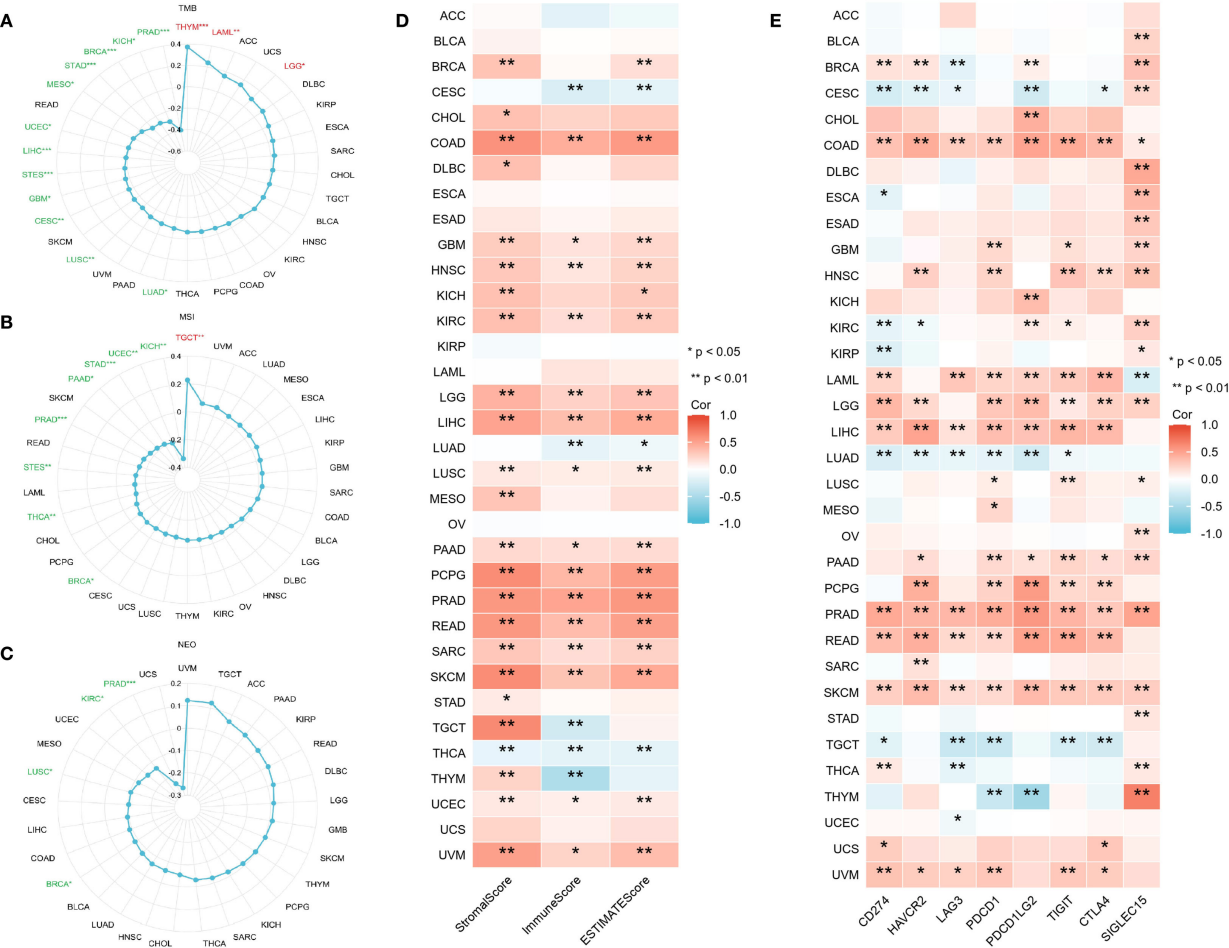
CLIC6 expression and tumor immunogenomic features. **(A–C)** Correlation with TMB, MSI, and NEO. **(D)** Association with TME scores. **(E)** Link to immune checkpoint expression across 34 cancers (*P<0.05, **P<0.01, ***P<0.001).

Earlier research indicates that TMB, MSI, and NEO can predict how cancer patients will respond to immunotherapy, making them useful biomarkers for assessing the effectiveness of tumor treatments (24). This study investigated the correlation between CLIC6 mRNA expression levels and TMB, MSI, and NEO. Radar plots showed that CLIC6 expression was negatively correlated with TMB in 12 cancer types (PRAD, KICH, BRCA, STAD, MESO, UCEC, LIHC, STES, GBM, CESC, LUSC, LUAD) and positively correlated with TMB in THYM, LAML, and LGG (*P* < 0.05; **Figure 8A**). Additionally, among nine cancer types, CLIC6 expression correlates with MSI, showing negative correlations in KICH, UCEC, STAD, PAAD, PRAD, STES, THCA, and BRCA, and a positive correlation in TGCT (*P* < 0.05; **Figure 8B**). Similarly, in PRAD, KIRC, LUSC, and BRCA, CLIC6 expression negatively correlated with NEO (*P* < 0.05; **Figure 8C**).

The study further examined the association between CLIC6 mRNA expression levels and the scores for tumor stroma, immune infiltration, and tumor purity across various cancer types. Heatmap analyses revealed a positive correlation between CLIC6 mRNA expression levels and the scores for tumor stroma, immune proliferation, and tumor purity in 15 distinct cancer types (COAD, GBM, HNSC, KIRC, LGG, LIHC, LUSC, PAAD, PCPG, PRAD, READ, SARC, SKCM, UCEC, and UVM) and negatively correlated with THCA (*P* < 0.05; **Figure 8D**). Further analysis was conducted to investigate the correlation between CLIC6 mRNA expression levels and immune checkpoint genes (CD274, HAVCR2, LAG3, PDCD1, PDCD1LG2, TIGIT, CTLA4, and SIGLEC15). The heatmap showed that CLIC6 expression showed positive interactions with most immune checkpoints in COAD, HNSC, LAML, LGG, LIHC, PAAD, PCPG, PRAD, READ, SKCM, and UVM, with COAD, PRAD, and SKCM showing positive correlations with all immune checkpoints; whereas in CESC, LUAD, and TGCT, CLIC6 expression showed negative correlations with most immune checkpoints (*P* < 0.05; **Figure 8E**).

**Figure 9:**
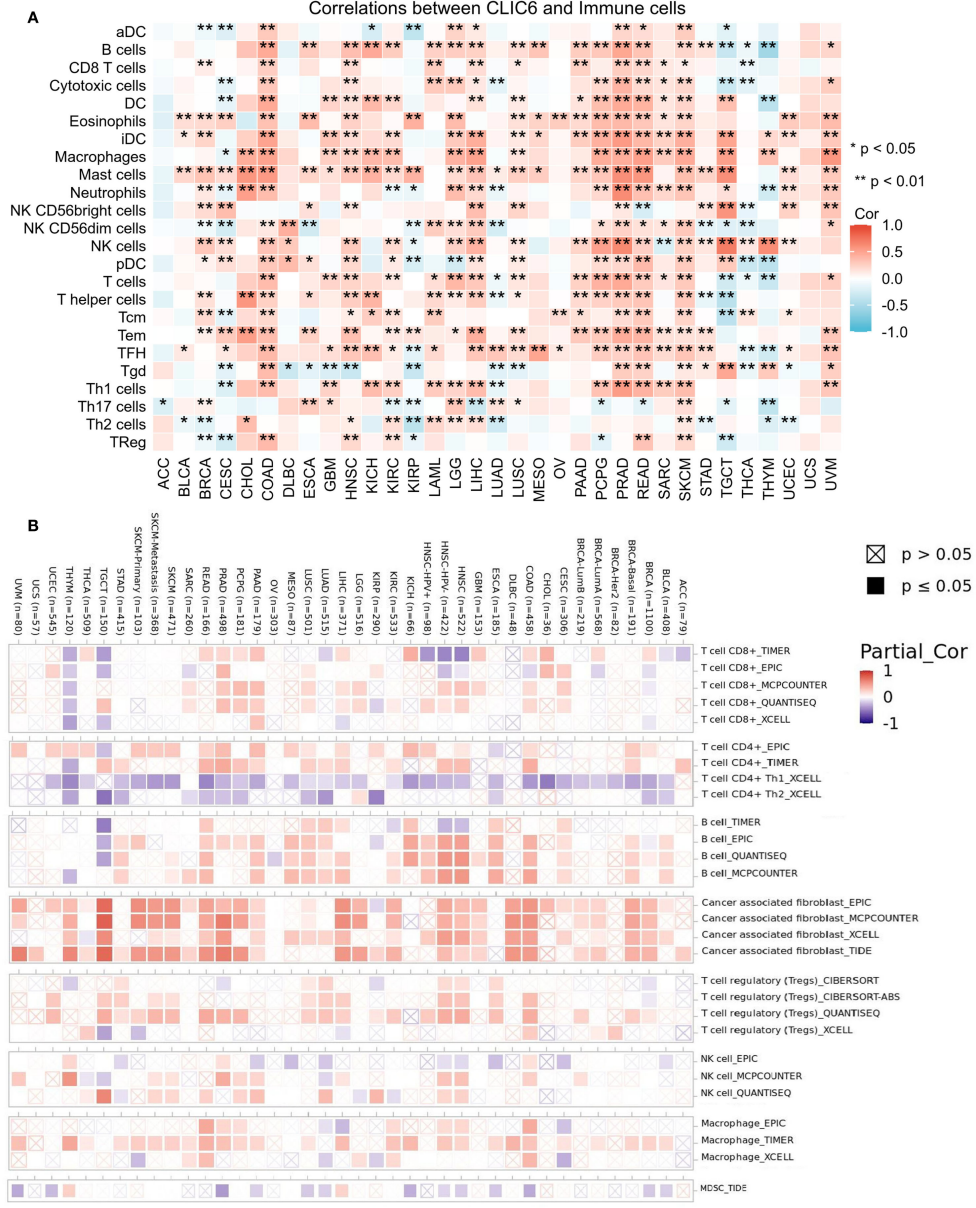
CLIC6 expression and immune cell infiltration in pan-cancer. **(A)** ssGSEA-based correlation with immune infiltration. **(B)** TIMER2.0 analysis of CLIC6 association with CAF infiltration (*P<0.05, **P<0.01).

Tumor-infiltrating immune cells (TIICs), as integral elements of the tumor microenvironment (TME), are pivotal in the therapeutic management of cancer. Therefore, the ssGSEA method was employed in this study to assess the relationship between CLIC6 mRNA expression levels and the infiltration levels of 24 different types of TIICs. Heatmaps showed that in 14 cancer types, including BRCA, COAD, HNSC, KIRC, LGG, LIHC, LUSC, PAAD, PCPG, PRAD, READ, SARC, SKCM, and UVM, CLIC6 mRNA expression levels were positively correlated with the infiltration levels of most TIICs, with some TIICs exhibiting a significant positive correlation between their infiltration levels and CLIC6 mRNA expression levels. such as CD8+ T cells, dendritic cells (DC), eosinophils, immature DC cells, macrophages, mast cells, natural killer cells, effector memory T cells, and follicular helper T cells (**Figure 9A**). Additionally, this study employed the Timer 2.0 database alongside the CIBERSORT, quanTIseq, xCell, MCP-counter, and EPIC algorithms to examine the relationship between CLIC6 mRNA expression levels and the infiltration of various TIICs. The findings indicated a positive correlation between CLIC6 mRNA expression levels and the infiltration levels of cancer-associated fibroblasts in the majority of cancer types (**Figure 9B**).

### 4.7 Mechanism of action of CLIC6 in breast cancer and pan-cancer

**Figure 10:**
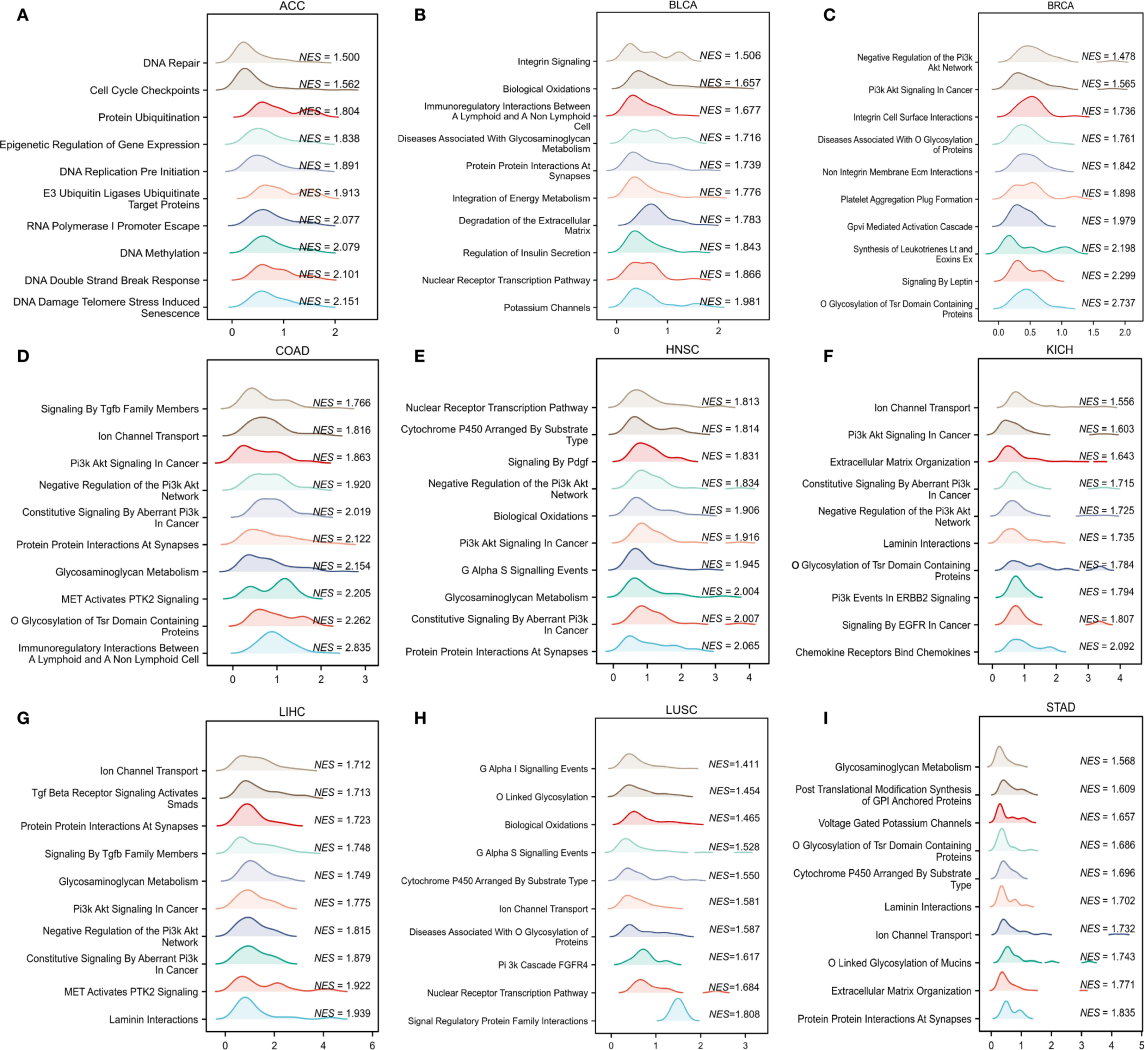
GSEA functional enrichment analysis of CLIC6 in nine cancers. In ACC **(A)**, BLCA **(B)**, BRCA **(C)**, COAD **(D)**, HNSC **(E)**, KICH **(F)**, LIHC **(G)**, LUSC **(H)**, and STAD **(I)**, the first 10 pathways are positively related to CLIC6 expression.

To determine the possible mechanisms by which CLIC6 is involved in pan-cancer, this study focused on the selection of nine distinct cancer types (ACC, BLCA, BRCA, COAD, HNSC, KICH, LIHC, LUSC, STAD) for GSEA analysis. The findings indicated that genes exhibiting a positive correlation with CLIC6 expression were predominantly enriched in pathways related to ion channel transport, nuclear receptor-mediated transcription, glycosaminoglycan metabolism, protein interactions within the synapse, and the PI3K/AKT signaling pathway (**Figure 10**). Genes negatively correlated with CLIC6 expression were enriched in DNA replication, DNA or protein methylation, glycolysis, cellular senescence or apoptosis, and the TP53 pathway (**Supplementary Figure S5**).

**Figure 11:**
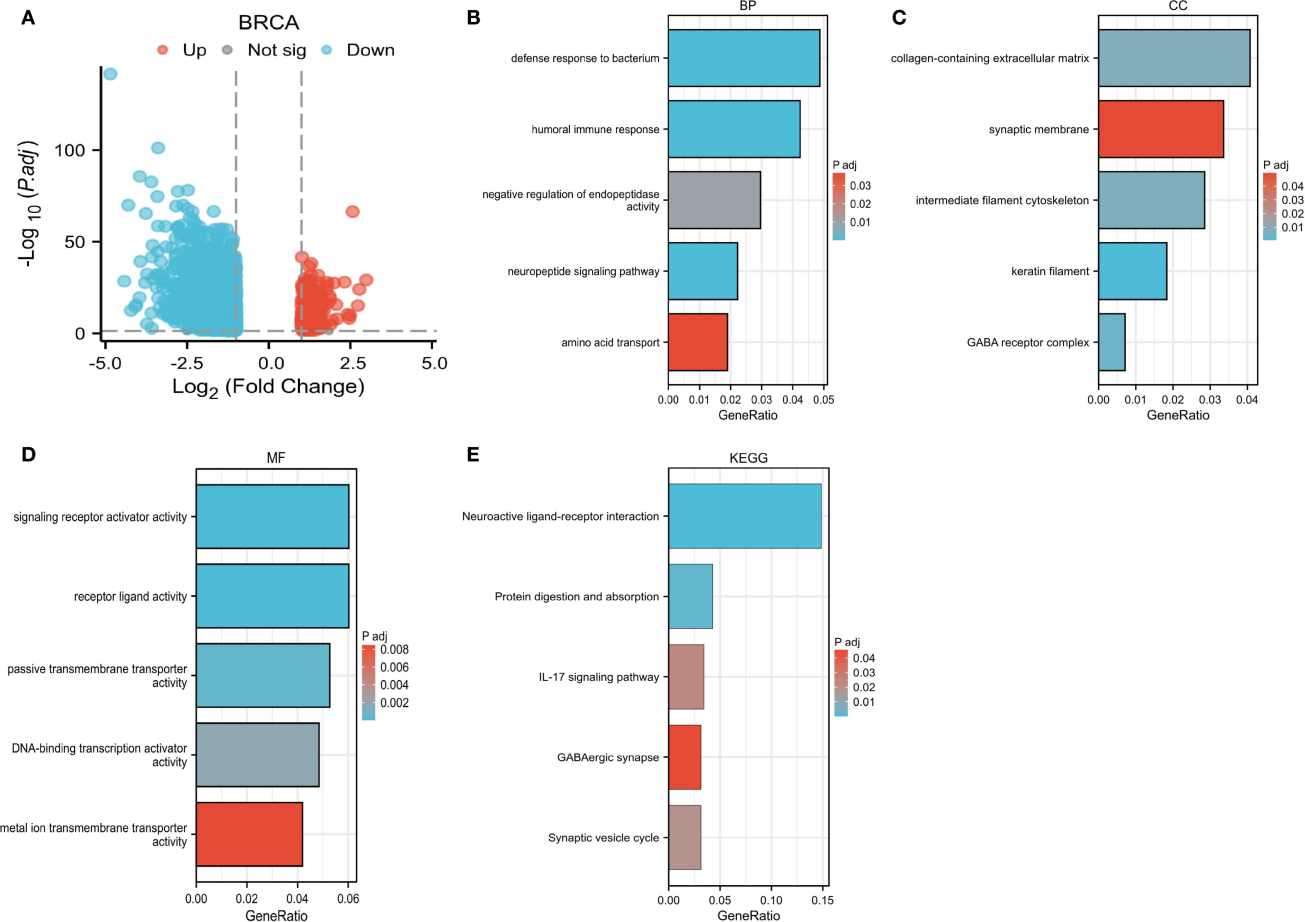
CLIC6 interactions and functional enrichment. **(A)** Volcano map of CLIC6. **(B-E)** GO and KEGG pathway analysis.

Moreover, this study conducted an in-depth investigation into the potential pathways through which CLIC6 may contribute to the development of breast cancer. It also identified proteins that interact with CLIC6 and carried out an enrichment analysis. A volcano plot was used to visually display the upregulation, downregulation, and significance of proteins interacting with CLIC6 in breast cancer (**Figure 11A**). Subsequently, GO/KEGG enrichment analysis was performed using the aforementioned CLIC6-interacting proteins, identifying eight KEGG pathways and 110 GO categories, including 45 biological processes (BP), 11 cellular components (CC), and 54 molecular functions (MF) (**Supplementary Table S3**). The five most prominent cancer-associated terms within each GO category were emphasized. The GO analysis results indicated that CLIC6 primarily participates in biological processes such as defense against bacteria, humoral immune response, negative regulation of endopeptidase activity, neuropeptide signaling pathways, and amino acid transport (**Figure 11B**); Furthermore, it contributes to the formation of cellular components, including the collagen-containing extracellular matrix, synaptic membranes, intermediate filament cytoskeleton, keratin fibers, and GABA receptor complexes (**Figure 11C**); additionally, CLIC6 is implicated in receptor–ligand interactions, signal receptor activation, passive transmembrane transport, DNA-binding transcription activation, and metal ion transmembrane transport (**Figure 11D**). KEGG analysis suggests that CLIC6 may exert its effects through mediating neuroactive ligand–receptor interactions, the IL-17 signaling pathway, protein digestion and absorption, synaptic vesicle recycling, and GABAergic synapses (**Figure 11E**).

### 4.8 Expression of CLIC6 in BRCA tissues and regulation of cancer cell phenotype

**Figure 12:**
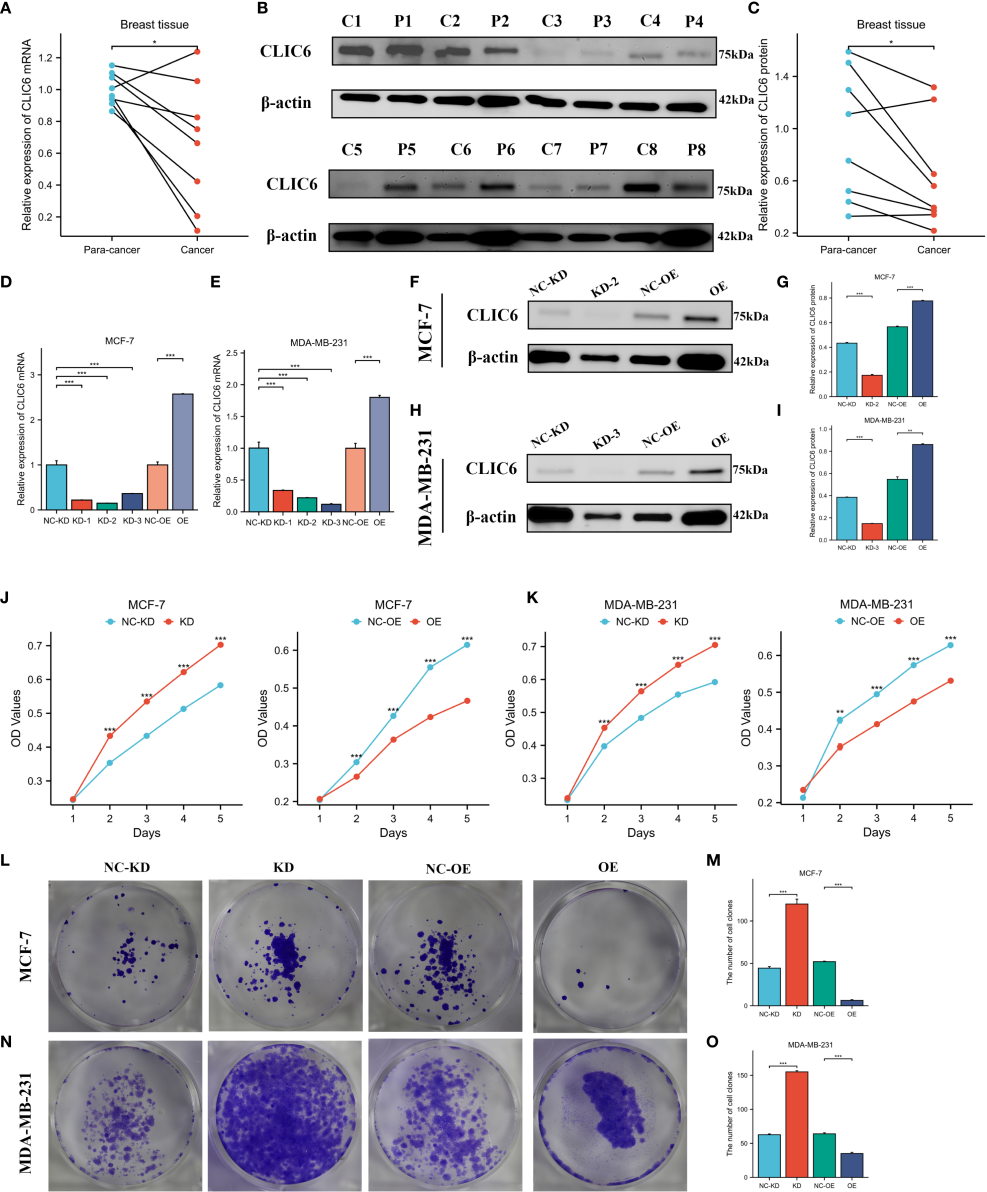
Effect of CLIC6 on breast cancer proliferation capacity. **(A)** qRT-PCR analysis of CLIC6 expression in paired breast cancer tissues (n=8) and matched adjacent normal tissues. Values are presented as mean ± standard deviation, normalized to GAPDH, p<0.01. **(B, C)** Western blot analysis of CLIC6 expression in paired breast cancer tissues (n=8) and matched adjacent normal tissues. **(D, E)** qRT-PCR analysis validating CLIC6 knockdown and overexpression efficiency in MCF-7 and MDA-MB-231 cell lines. **(F-I)** Western blot validation of CLIC6 knockdown and overexpression efficiency in cells. β-actin served as loading control. Molecular weight markers (kDa) are shown on the right. Bar graphs display optical density quantification results from three biological replicates (p<0.01). **(J, K)** CCK8 assay verifying the effect of different CLIC6 expression levels on the proliferation capacity of breast cancer cell lines. **(L-O)** Colony formation assays of MCF-7 and MDA-MB-231 cells with different CLIC6 expression levels. Representative images are shown. The right panel displays the mean colony count ± standard deviation from three independent experiments. *P* < 0.01. *P<0.05 , **P< 0.01, ***P<0.001.

**Figure 13:**
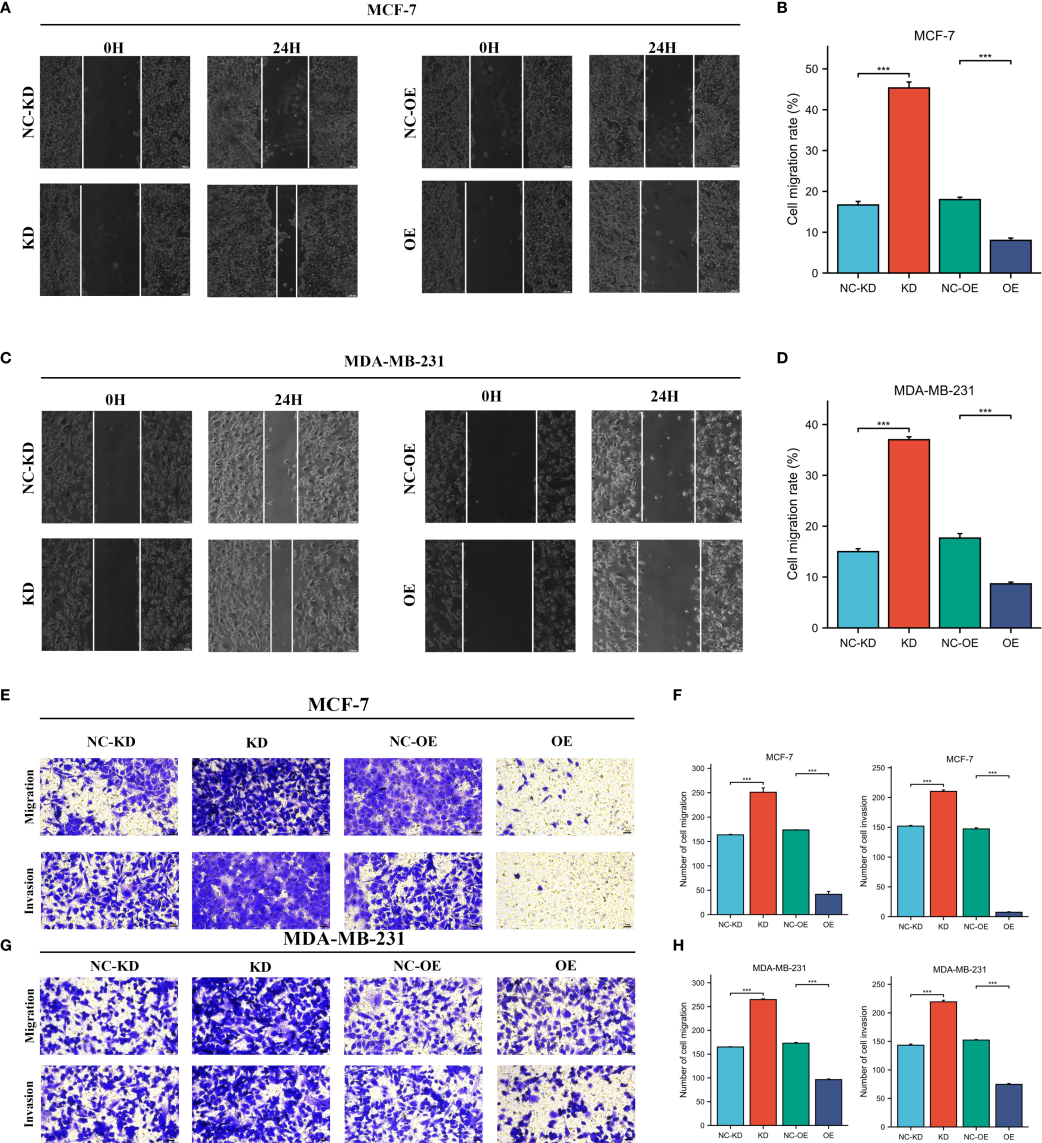
Effect of CLIC6-KD and CLIC6-OE on BRCA invasion and migration. **(A-D)** Scratch assay: The effects of CLIC6-KD and CLIC6-OE on the migration ability of BRCA cell lines. **(E-H)** Transwell invasion and migration assay: Changes in BRCA cell lines invasion and migration after CLIC6-KD and CLIC6-OE. ***p<0.001.

The expression levels of CLIC6 were validated using cancerous and adjacent tissue samples from eight breast cancer patients. The findings from the qRT-PCR and WB analyses demonstrated that CLIC6 was downregulated in breast cancer tissue (**Figures 12A-C**). To validate the regulatory role of different CLIC6 expression levels on BRCA cell phenotypes, CLIC6 knockdown (CLIC6-KD) and overexpression (CLIC6-OE) BRCA cell lines were established and validated by qRT-PCR and WB (**Figures 12D-I**). CCK-8 assay results showed that CLIC6-OE significantly inhibited BRCA cell proliferation, while CLIC6-KD had the opposite effect (**Figures 12J, K**). Cell cloning assay demonstrated that knockdown of CLIC6 promoted clonogenicity in BRCA cell lines, whereas overexpression inhibited it (**Figures 12L-O**). Scratch assays showed that silencing CLIC6 increased MCF-7 cell migration, while overexpressing it reduced their migration. The same effects were observed in MDA-MB-231 cells (**Figures 13A-D**). Additionally, the transwell assays showed that CLIC6-OE greatly reduced the invasion and migration abilities of BRCA cell lines. Conversely, CLIC6-KD enhanced these cellular processes (**Figures 13E-H**). In summary, high CLIC6 expression greatly reduces proliferation, invasion, and migration in BRCA cell lines, whereas low expression enhances these activities.

## 5 Discussion

The CLIC family has multiple members, and CLIC6 is one of them. Among them, CLIC1 is closely related and has the clearest functional characteristics. CLIC1 is regarded as a potential biomarker and therapeutic target for tissues, blood, and interstitial fluid. In breast cancer, Xia (25) found that the expression of CLIC1 was increased at both RNA and protein levels. The overexpression of CLIC1 was closely related to tumor size, TNM classification, pathological grade, lymph node metastasis, and Ki67. In colorectal cancer, Petrova (26) found that the CLIC1 protein is significantly overexpressed in cancer tissues and indicate that CLIC1 is a biomarker for colorectal cancer. In addition, CLIC1 has been widely confirmed to play a significant role in the progression and metastasis of various cancers. While CLIC6 has been linked to cancer progression, its comprehensive role across various cancers is not well comprehended. This study employs bioinformatics methods based on multiple datasets to systematically reveal the clinical significance and potential functional mechanisms of CLIC6 in different cancer types.

In the course of this research, an analysis of expression differences was performed on both paired and unpaired samples from 33 types of malignant tumors utilizing the TCGA and GTEx databases. The findings showed that CLIC6 expression exhibited variability across different cancer types, with a predominantly low expression observed in the majority of cases. Subsequently, this study investigated the prognostic significance of CLIC6 across various cancer types. Notably, the study discovered high CLIC6 expression in LAML, KIRP, and LUAD. For LAML patients, high CLIC6 expression is a risk factor for poor prognosis, whereas for KIRP and LUAD patients, high CLIC6 expression is a protective factor for better prognosis. CLIC6 is lowly expressed in BRCA, LGG, HNSC, STAD, LUSC, and PRAD, and low CLIC6 expression is a risk factor for poorer prognosis in BRCA, HNSC, LUSC, and PRAD patients; conversely, low CLIC6 expression predicts better outcomes in LGG and STAD patients. Aligned with this study’s findings, Liu (27) demonstrated that elevated CLIC6 expression in breast cancer patients is associated with improved survival outcomes compared to those with lower expression levels, indicating its role as a protective factor. Similarly, Zhai (28) also reached similar conclusions from their study on prostate cancer. This study also found that CLIC6 expression was higher in early clinical pathological stages than in advanced stages among patients with BRCA, KICH, KIRP, LUAD, and THCA, suggesting that CLIC6 may influence the prognosis of these cancer patients. Furthermore, CLIC6 could be a diagnostic biomarker for various cancers, and this study found that CLIC6 may act as an independent factor influencing the prognosis of patients with BRCA, LUAD, STAD, and LGG, offering a theoretical foundation for its prospective application in the treatment and management of cancer. In summary, CLIC6 appears to play distinct roles in different cancers, thereby exerting varying effects on patient prognosis.

Next, this study investigated the diagnostic significance of CLIC6 across various cancer types, revealing that CLIC6 demonstrates substantial predictive potential in the majority of these malignancies. Among them, CLIC6 has the best diagnostic effect in SKCM. However, there is a lack of studies, highlighting the need for more research to explore CLIC6’s potential as a cancer diagnostic marker.

Epigenetics refers to changes in gene expression that occur without altering the DNA sequence of the gene itself, through chemical modifications or other molecular processes (29). Epigenetic modifications serve as crucial regulatory elements in tumorigenesis and tumor progression and are implicated in diverse biological activities of tumor cells, including proliferation, invasion, metastasis, and metabolic reprogramming (30, 31). DNA methylation is the most classical form of epigenetic modification, catalyzed by DNA methyltransferases, which add methyl groups to cytosines in CpG islands, thereby regulating gene expression. It is crucial for tumor spread and metastasis (32, 33). This study investigated the correlation between CLIC6 expression and CLIC6 promoter methylation levels, as well as the expression of m6A methylation-related regulatory factors, across different cancers. The results showed that the CLIC6 promoter exhibited high methylation levels in most tumors, consistent with most tumor suppressor genes, and CLIC6 promoter methylation levels were inversely related to CLIC6 mRNA expression. Additionally, in most malignant tumors, CLIC6 expression was positively linked to m6A methylation regulators, with a strong positive correlation observed with THYM, suggesting that CLIC6 may exhibit elevated m6A methylation levels in THYM. In summary, modifications in CLIC6 methylation are significant in the context of pan-cancer; however, additional functional experiments are required to substantiate its mechanistic role.

Epigenomic disruption is one of the core characteristics of cancer, as it alters cellular properties by regulating gene expression patterns and disrupts the dynamic balance between cells and the tumor microenvironment, thereby driving tumorigenesis and progression (34). This study found that CLIC6 mutations are present in certain types of cancer, and they are positively correlated with CLIC6 mRNA expression levels. Due to CLIC6 amplification mutations and high levels of CLIC6 mRNA expression, patients with various cancers have poorer prognoses. Therefore, this study speculates that CLIC6 amplification mutations are an important cause of elevated CLIC6 mRNA expression levels, resulting in poor outcomes for cancer patients. Thus, CLIC6 mutations are likely crucial in cancer development and progression. This study was the first to highlight the significant role of CLIC6 mutations in pan-cancer, but additional experimental investigations are required to elucidate the mechanisms through which CLIC6 mutations affect cancer development and progression.

Cancer immunotherapy boosts the immune system to help it target and destroy cancer cells. Immune checkpoint inhibitors (ICIs) are the main strategy in cancer immunotherapy (35). TMB and MSI are considered the main predictive biomarkers for ICI response, with elevated levels indicating that cancer patients are more sensitive to ICI treatment and have more significant survival benefits (36, 37). This study investigated the relationship between CLIC6 expression and TMB, MSI, and NEO. The findings indicated a significant negative correlation between CLIC6 and TMB, MSI, and NEO in patients with PRAD and BRCA. CLIC6 mRNA was downregulated in PRAD and BRCA, indicating that low CLIC6 expression is associated with higher TMB, MSI, and NEO scores in PRAD and BRCA patients, suggesting that PRAD and BRCA patients with lower CLIC6 expression may benefit more from immunotherapy. Additionally, the study discovered a positive correlation between CLIC6 expression and both immunological scores and immune checkpoint expression in COAD, HNSC, LGG, LIHC, PRAD, READ, and SKCM. Zhou (38) found that CLIC6 can exert potent antitumor effects in liver cancer by regulating cytokine levels and immune cell balance, consistent with the findings of this study. However, this study found that CLIC6 exhibits low expression levels in these tumors, suggesting it may negatively impact patient prognosis by reducing tumor immune scores and suppressing the activation of immune-related genes.

Subsequently, this study examined the relationship between CLIC6 expression and immune cell infiltration in the tumor microenvironment across different cancer types. The results revealed a positive correlation between CLIC6 mRNA expression and the infiltration of immune cells that participate in antitumor activity, such as dendritic cells (DCs), eosinophils, macrophages, mast cells, NK cells, Th cells, and follicular helper T (FHL) cells. These immune cells contribute to tumor immunity through mechanisms including the secretion of diverse cytokines and chemokines, as well as antigen presentation, thus facilitating both innate and adaptive immune responses (39). CLIC6 mRNA is lowly expressed in most tumors, so this study speculates that the absence of CLIC6 may lead to the loss of antitumor immune effects rather than through mediating immune escape or immune suppression. In summary, this study suggests that CLIC6 may regulate tumor immune effects, making it a potential target for novel tumor immunotherapy.

To investigate the potential mechanisms and functions of CLIC6 across various cancer types, this study performed GSEA on selected malignancies. The findings indicated a positive correlation between CLIC6 and ion channel transport, nuclear receptor transcription pathways, glycosaminoglycan metabolism, protein interactions in protein synapses, and the PI3K/AKT signaling pathway. Ion channels facilitate cancer progression and metastasis by affecting tumor-related cellular behaviors like proliferation, apoptosis, migration, and angiogenesis (40). Ko (41) found that the ion channel IC30, which includes CLIC6, is associated with P53 mutation status, ER status, and histological tumor grade in breast cancer, making it a promising breast cancer diagnostic and prognostic biomarker. consistent with the results of this study. Furthermore, this study conducted GO and KEGG analyses to investigate CLIC6’s potential roles in BRCA. The outcome showed that CLIC6 is involved in regulating immune responses. Combined with its expression levels in BRCA, this further validated the hypothesis that the absence of CLIC6 expression is associated with the loss of anti-tumor immune effects. At the same time, our cell experiments verified that overexpression of CLIC6 can inhibit BRCA cell proliferation, invasion, and migration and can promote apoptosis. In summary, this study offers a novel perspective on the role of CLIC6, contributing to the advancement of innovative cancer treatment strategies.

## 6 Conclusion

This study represents the inaugural systematic investigation into CLIC6 expression, prognosis, diagnosis, epigenetics, methylation, immunological analysis, and enrichment analysis across various cancers utilizing bioinformatics methodologies. The results suggest that CLIC6 could be used as a prognostic indicator and treatment target in cancerous tumors. In conclusion, these findings better understand CLIC6’s role in cancer and suggest new avenues for innovative immunotherapy strategies.

## Data Availability

The original contributions presented in the study are included in the article/**Supplementary Material**. Further inquiries can be directed to the corresponding author.

## Glossary

BLCA: bladder urothelial carcinoma
BRCA: breast cancer
CESC: cervical squamous cell carcinoma and endocervical adenocarcinoma
CHOL: cholangiocarcinoma
CNV: copy number variants
COAD: colon adenocarcinoma
CLIC6-KD: CLIC6 knockdown
CLIC6-OE: CLIC6 overexpression
DLBC: lymphoid neoplasm diffuse large B-cell lymphoma
DSS: disease-specific survival
ESCA: esophageal carcinoma
GBM: glioblastoma multiforme
HNSC: head and neck squamous cell carcinoma
IHC: immunohistochemistry
KICH: kidney chromophobe cell carcinoma
KIRC: kidney renal clear cell carcinoma
KIRP: kidney renal papillary cell carcinoma
K-M: Kaplan–Meier
LAML: acute myeloid leukemia
LGG: brain lower grade glioma
LIHC: liver hepatocellular carcinoma
LUAD: lung adenocarcinoma
LUSC: lung squamous cell carcinoma
MSI: microsatellite instability
NEO: neoantigen
OS: overall survival
OV: ovarian serous cystadenocarcinoma
PAAD: pancreatic adenocarcinoma
PPI: protein–protein interaction
PFS: progression-free survival
PRAD: prostate adenocarcinoma
READ: rectum adenocarcinoma
SKCM: skin cutaneous melanoma
STAD: stomach adenocarcinoma
TGCT: testicular germ cell tumors
THYM: thymoma
TMB: tumor mutational burden
TME: tumor microenvironment
UCEC: uterine corpus endometrial carcinoma
UCS: uterine carcinosarcoma
WB: western blotting
