# Supplementary material for: CLIC6’s role in cancer: from broad analysis to breast cancer validation
Source: Front Oncol. 2025 Oct 9;15:1667589. doi: 10.3389/fonc.2025.1667589 (PMC12545143; doi:10.3389/fonc.2025.1667589)
Supplement: Supplementary file 1 [file DataSheet1.pdf]

Supplementary materials for

CLIC6's role in cancer: from broad analysis to breast cancer validation

Junyi Wang[1†], Yiyang Wang[1†], Haotian Ma[1†], Yongxiang Li[1], Jiayue Hou[2], Jiaqi Li[2], Dilimulati Ismtula[1*], Chenming Guo[1,3*]

[1]Department of Breast Surgery, Center of Digestive and Vascular, The First Affiliated Hospital of Xinjiang Medical University, Urumqi 830054, China;

[2]Clinical Medicine Department, Xinjiang Medical University, Urumqi 830054, China;

[3]The First Affiliated Hospital of Xinjiang Medical University, State Key Laboratory o f Pathogenesis, Prevention, Treatment of Central Asian High Incidence Diseases in C entral Asia;

[*]Corresponding author

†These authors made equal contributions to this work and shared the first authorship.

[*]Correspondence: Chenming Guo, gcm_xjmu@yeah.net. Dilimulati Ismtula, mlt0306 @sina.com.

Additional material for this article can be found in the Supplementary Graphics and Table Legend module.

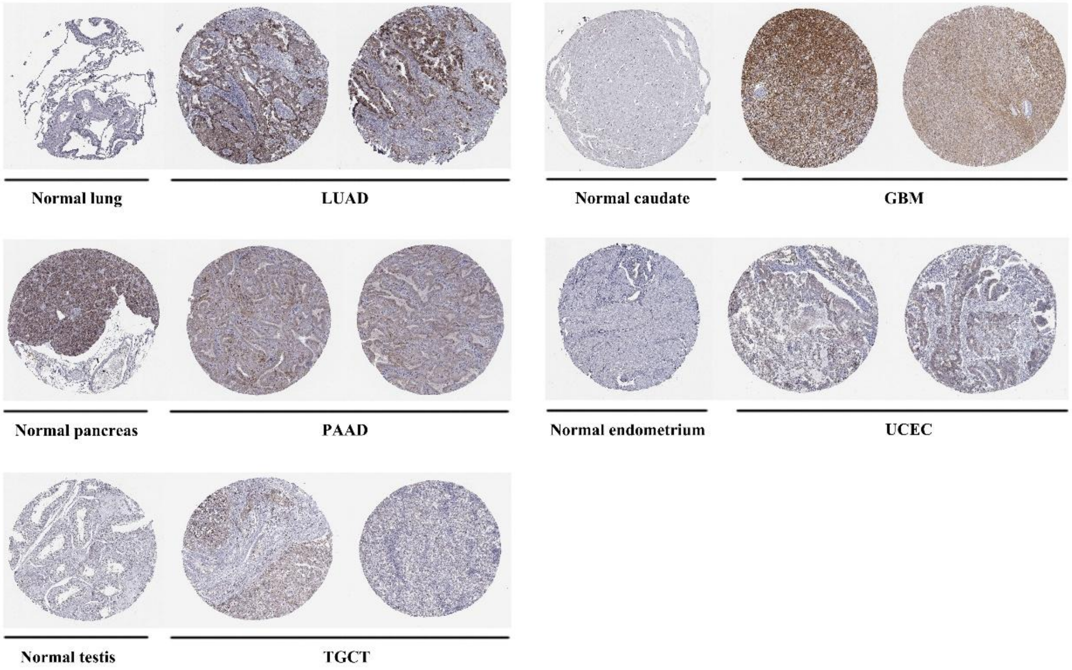

Supplementary Figure S1. Immunohistochemical Staining of 5 Normal Tissues and Tumor Tissues from the HPA Database. (Antibody: HPA065285, 100x magnification)

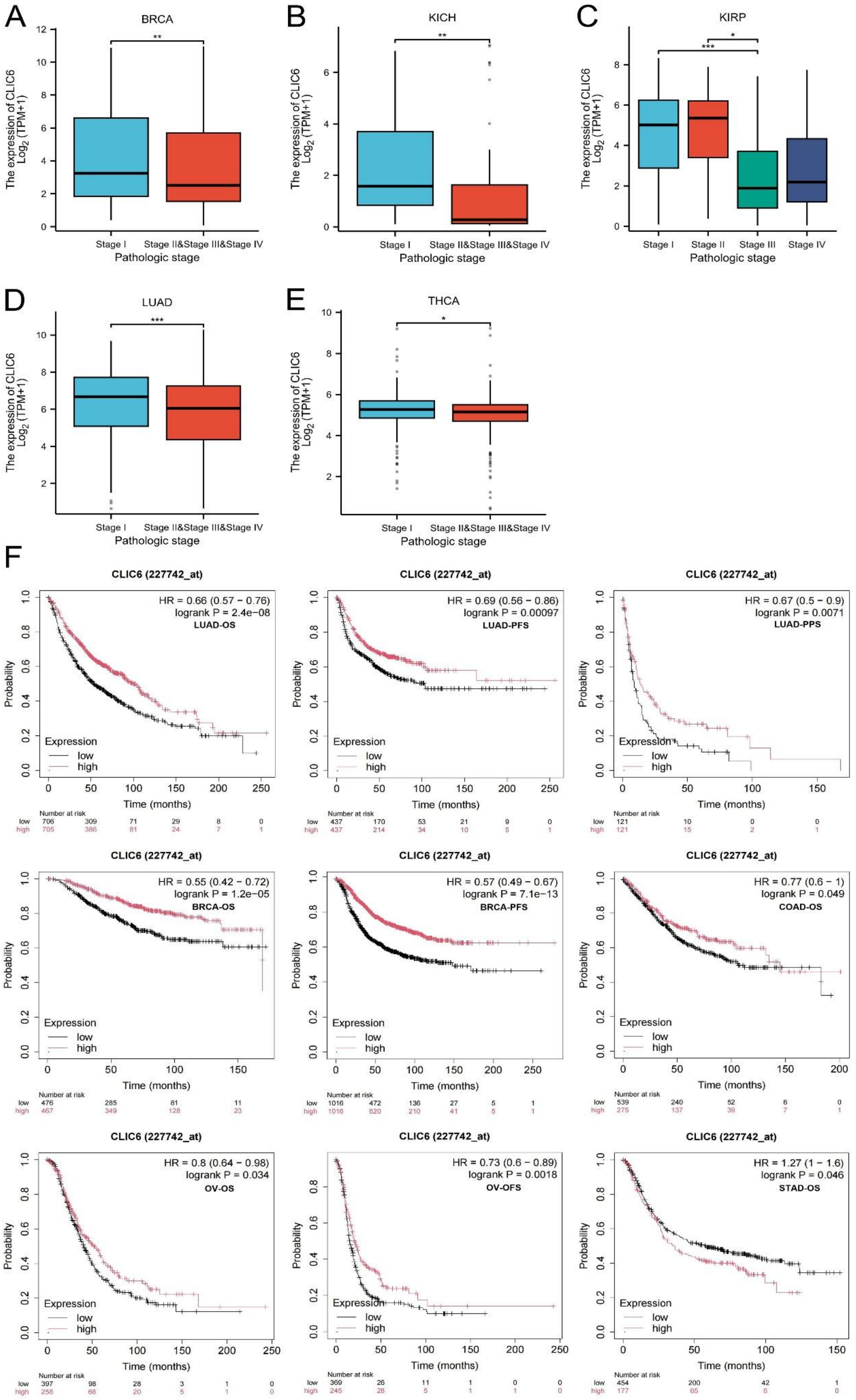

A
BRCA
**
The expression of CLIC6 Log2 (TPM+1)
Stage I
Stage II&Stage III&Stage IV
Pathologic stage
B
KICH
**
C
KIRP
***
*
Stage I
Stage II
Stage III
Stage IV
D
LUAD
***
E
THCA
*
F
CLIC6 (227742_at)
HR = 0.66 (0.57 − 0.76)
logrank P = 2.4e−08
LUAD-OS
HR = 0.69 (0.56 − 0.86)
logrank P = 0.00097
LUAD-PFS
HR = 0.67 (0.5 − 0.9)
logrank P = 0.0071
LUAD-PPS
HR = 0.55 (0.42 − 0.72)
logrank P = 1.2e−05
BRCA-OS
HR = 0.57 (0.49 − 0.67)
logrank P = 7.1e−13
BRCA-PFS
HR = 0.77 (0.6 − 1)
logrank P = 0.049
COAD-OS
HR = 0.8 (0.64 − 0.98)
logrank P = 0.034
OV-OS
HR = 0.73 (0.6 − 0.89)
logrank P = 0.0018
OV-OFS
HR = 1.27 (1 − 1.6)
logrank P = 0.046
STAD-OS
Probability
Time (months)
Expression
low
high
Number at risk

Supplementary Figure S2. (A-E) The relationship between CLIC6 expression levels and pathological characteristics in BRCA, KICH, KIRP, LUAD, and THCA. **(F)** K-Mplotter database showing the association between CLIC6 expression and prognosis in cancer patients with BRCA, LUAD, COAD, STAD, and OV.

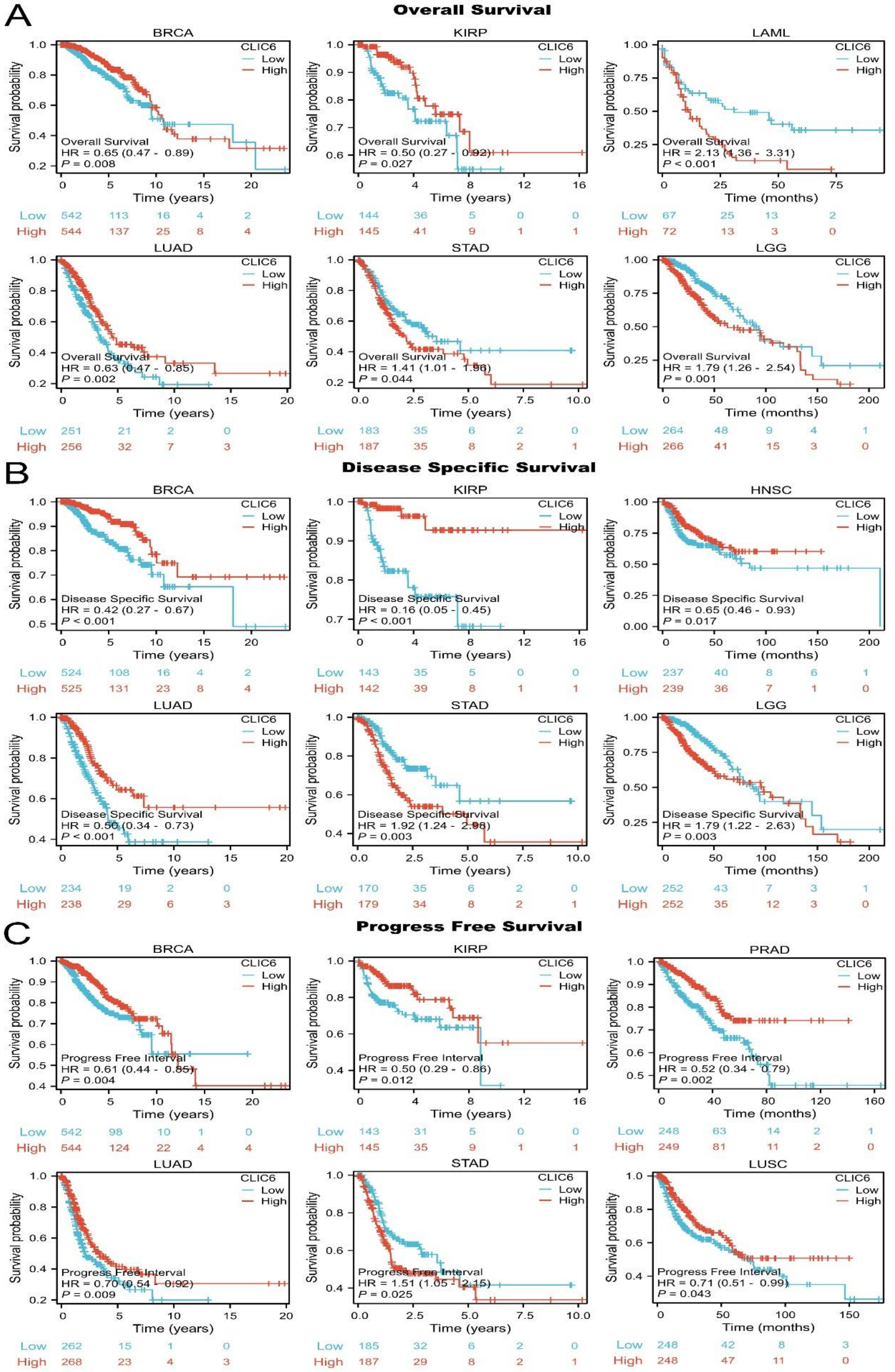

Supplementary Figure S3. Kaplan-Meier (KM) Curves Showing the Detailed Association Between CLIC6 Expression and Prognosis in Cancer Patients. **(A)** Overall Survival (OS) in BRCA, KIRP, LAML, LUAD, STAD and LGG. **(B)** Disease-Specific Survival (DSS) in BRCA, KIRP, HNSC, LUAD, STAD and LGG. **(C)** Progression-free survival (PFS) in BRCA, KIRP, PRAD, LUAD, STAD, and LUSC.

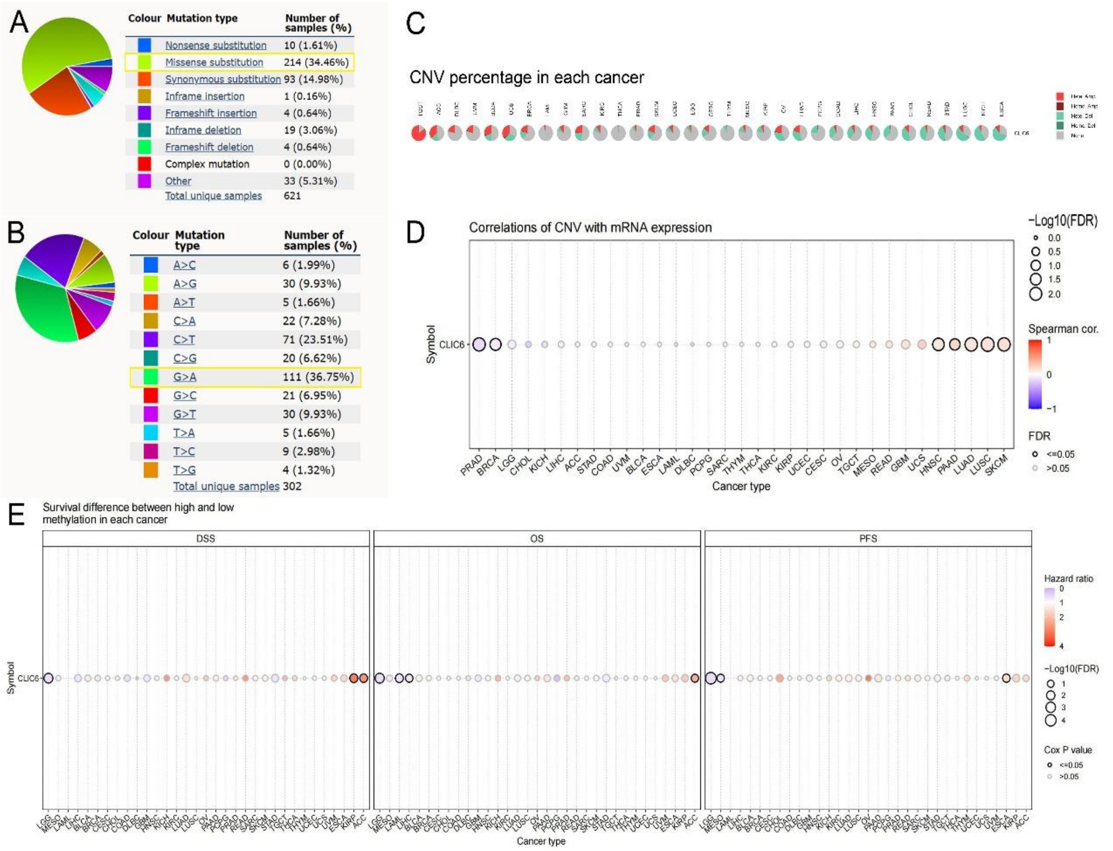

Supplementary Figure S4. Mutation analysis and epigenetic methylation analysis of CLIC6. **(A)** The main mutation types of CLIC6. **(B)** The main types of single nucleotide variants (SNVs) in CLIC6. **(C)** Percentage of CLIC6 copy number variants (CNVs) in each cancer. **(D)** Correlation between CLIC6 expression and CNVs. **(E)** Effect of CLIC6 methylation levels on prognosis in cancer patients.

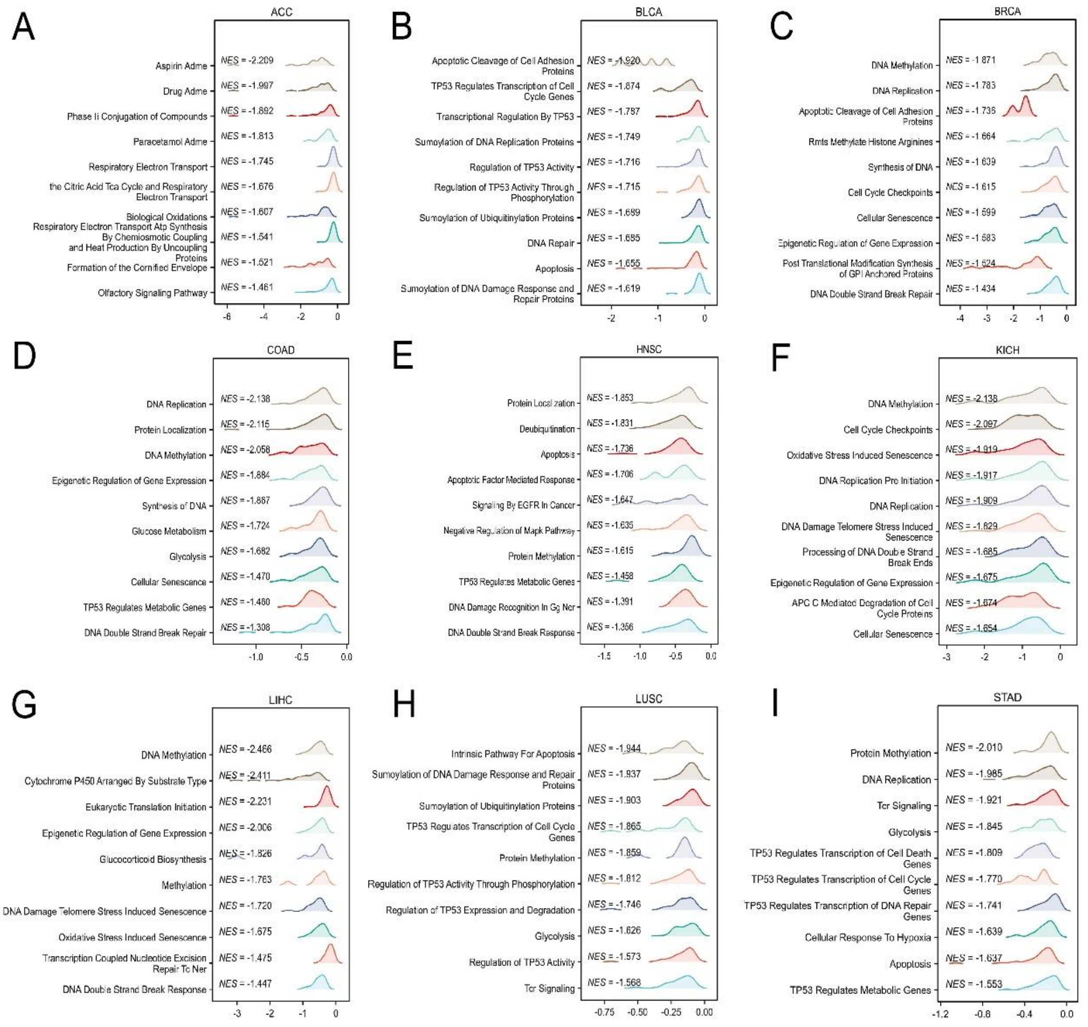

Supplementary Figure S5. GSEA functional enrichment analysis of CLIC6 in nine types of cancer. In ACC **(A)**, BLCA **(B)**, BRCA **(C)**, COAD **(D)**, HNSC **(E)**, KICH **(F)**, LIHC **(G)**, LUSC **(H)**, and STAD **(I)**, the top ten pathways were negatively correlated with CLIC6 expression.

Supplementary Table S1. CLIC6 shRNA sequences and primer sequences.

Name	Targeting sequence (5'→3')
sh-CLIC6-1	GCAAGCTGAGAAGTTCGTGA
sh-CLIC6-2	GGTGAAGATGCTGAACTCTT
sh-CLIC6-3	GCTGAGAAGTTCGTGAGTGT
CLIC6-OE-F	AAGCTTATGAGCCTCGGCCGCCTT
CLIC6-OE-R	GGTACCTCATGAGTGCCGGTGGAA
CLIC6-F	GGGACCCAACATCCCGAATC

CLIC6-R	TCAGGCAGAGGGCTATTTAAGT
GAPDH-F	GAAGGTGAAGGTCGGAGTC
GAPDH-R	GAAGATGGTGATGGGATTTC

Supplementary Table S2. Results of univariate and multivariate Cox analysis of clinical parameters in BRCA **(A)**, LUAD **(B)**, STAD **(C)**, LGG **(D)**.

A. BRCA

Characteristics	Total(N)	Univariate analysis		Multivariate analysis	
		Hazard ratio (95% CI)	P value	Hazard ratio (95% CI)	P value
Pathologic M stage	925				
M0	905	Reference		Reference	
M1	20	4.266 (2.474 - 7.354)	**< 0.001**	2.549 (1.320 - 4.926)	**0.005**
Pathologic stage	1,062				
Stage I&Stage II	800	Reference		Reference	
Stage III&Stage IV	262	2.367 (1.686 - 3.321)	**< 0.001**	2.181 (1.478 - 3.220)	**< 0.001**
Age	1,086				
<= 60	603	Reference		Reference	
> 60	483	2.024 (1.468 - 2.790)	**< 0.001**	1.989 (1.388 - 2.849)	**< 0.001**
PR status	1,033				
Negative	342	Reference			
Positive	691	0.729 (0.521 - 1.019)	0.065		
ER status	1,036				
Negative	240	Reference			
Positive	796	0.709 (0.493 - 1.019)	0.063		
HER2 status	717				
Negative	560	Reference			
Positive	157	1.593 (0.973 - 2.609)	0.064		
CLIC6	1,086				
Low	542	Reference		Reference	
High	544	0.649 (0.470 - 0.894)	**0.008**	0.693 (0.490 - 0.981)	**0.038**

B. LUAD

Characteristics	Total(N)	Univariate analysis		Multivariate analysis	
		Hazard ratio (95% CI)	P value	Hazard ratio (95% CI)	P value
Pathologic T stage	527				
T1&T2	461	Reference		Reference	
T3&T4	66	2.352 (1.614 - 3.426)	**< 0.001**	1.874 (1.180 - 2.975)	**0.008**
Pathologic N stage	514				
N0	345	Reference		Reference	
N1&N2&N3	169	2.547 (1.904 - 3.407)	**< 0.001**	2.041 (1.388 - 3.002)	**< 0.001**
Pathologic M stage	381				
M0	356	Reference		Reference	
M1	25	2.176 (1.272 - 3.722)	**0.005**	1.318 (0.689 - 2.521)	0.404
Pathologic stage	522				
Stage I&Stage II	415	Reference		Reference	
Stage III&Stage IV	107	2.710 (1.994 - 3.685)	**< 0.001**	1.488 (0.915 - 2.421)	0.110
Gender	530				
Female	283	Reference			
Male	247	1.087 (0.816 - 1.448)	0.569		
Age	520				
<= 65	257	Reference			
> 65	263	1.216 (0.910 - 1.625)	0.186		
Smoker	516				
No	74	Reference			
Yes	442	0.942 (0.625 - 1.420)	0.775		
CLIC6	530				
Low	262	Reference		Reference	
High	268	0.668 (0.500 - 0.893)	**0.006**	0.642 (0.458 - 0.900)	**0.010**

C. STAD

Characteristics	Total(N)	Univariate analysis	Multivariate analysis

		Hazard ratio (95% CI)	P value	Hazard ratio (95% CI)	P value
Pathologic T stage	362				
T1&T2	96	Reference		Reference	
T3&T4	266	1.719 (1.131 - 2.612)	**0.011**	1.503 (0.815 - 2.773)	0.192
Pathologic N stage	352				
N0	107	Reference		Reference	
N1&N2&N3	245	1.925 (1.264 - 2.931)	**0.002**	1.377 (0.702 - 2.700)	0.352
Pathologic M stage	352				
M0	327	Reference		Reference	
M1	25	2.254 (1.295 - 3.924)	**0.004**	1.559 (0.754 - 3.226)	0.231
Pathologic stage	347				
Stage I&Stage II	160	Reference		Reference	
Stage III&Stage IV	187	1.947 (1.358 - 2.793)	**< 0.001**	1.087 (0.590 - 2.003)	0.789
Primary therapy outcome	313				
PD&SD	80	Reference		Reference	
PR&CR	233	0.244 (0.168 - 0.354)	**< 0.001**	0.267 (0.179 - 0.397)	**< 0.001**
Gender	370				
Female	133	Reference			
Male	237	1.267 (0.891 - 1.804)	0.188		
Age	367				
<= 65	163	Reference		Reference	
> 65	204	1.620 (1.154 - 2.276)	**0.005**	1.754 (1.167 - 2.637)	**0.007**
CLIC6	370				
Low	183	Reference		Reference	
High	187	1.407 (1.010 - 1.961)	**0.044**	1.701 (1.136 - 2.547)	**0.010**

D. LGG

Characteristics	Total(N)	Univariate analysis		Multivariate analysis	

		Hazard ratio (95% CI)	P value	Hazard ratio (95% CI)	P value
WHO grade	468				
G2	223	Reference		Reference	
G3	245	3.023 (2.022 - 4.519)	**< 0.001**	2.748 (1.787 - 4.225)	**< 0.001**
Primary therapy outcome	460				
PD&SD	257	Reference		Reference	
PR&CR	203	0.198 (0.111 - 0.353)	**< 0.001**	0.209 (0.111 - 0.392)	**< 0.001**
Age	530				
<= 40	265	Reference		Reference	
> 40	265	2.898 (2.015 - 4.168)	**< 0.001**	2.881 (1.865 - 4.451)	**< 0.001**
CLIC6	530				
Low	264	Reference		Reference	
High	266	1.786 (1.257 - 2.537)	**0.001**	1.978 (1.299 - 3.012)	**0.001**

Supplementary Table S3. GO terms and KEGG pathways enriched in the analysis.
